# Efficacy of different traditional Chinese medicine decoctions in the treatment of ischemic stroke: a network meta-analysis

**DOI:** 10.3389/fphar.2024.1486458

**Published:** 2024-11-01

**Authors:** Baicheng Ning, Xiaoting Zhu, Xiaodong Wu, Weiyao Zhu, Runze Wang, Chang Qi, Mingquan Li

**Affiliations:** ^1^ College of Integrated Chinese and Western Medicine, Changchun University of Chinese Medicine, Changchun, China; ^2^ Department of Cardiology, Dujiangyan Traditional Chinese Medicine Hospital, Chengdu, China; ^3^ College of Traditional Chinese Medicine, Changchun University of Chinese Medicine, Changchun, China

**Keywords:** traditional Chinese medicine, network meta-analysis, ischemic stroke, Xuefu Zhuyu decoction, Huatan Tongluo Decoction

## Abstract

**Objective:**

Currently, traditional Chinese medicine (TCM) and its combinations are widely used in the treatment and rehabilitation of patients with ischemic stroke. However, current studies should mainly focus on the therapeutic effects of traditional Chinese medicines alone. This paper will employ a network meta-analysis to compare the efficacy of different TCM decoctions in the treatment of patients with ischemic stroke.

**Methods:**

Chinese and English databases including PubMed, Embase, Cochrane Library, and Web of Science were searched to collect randomized controlled trials of TCM decoctions in the treatment of patients with ischemic stroke (IS), with a search time frame until April 2024. A library of references was created using EndNote 21. Quality assessment was performed using the Version 2 of the Cochrane risk-of-bias tool for randomized trials (RoB 2). A Bayesian network meta-analysis of data was performed using R4.3.1 and STATA 15.0.

**Results:**

A network meta-analysis was conducted on 119 randomized controlled trials including 12,137 IS patients. The following TCM decoctions were involved: Xinglou Chengqi Decoction (XLCQT), Shenqi Tongluo Decoction (SQTLF), Zhongfeng Jiuxian Decoction (ZFJXT), Yiqi Tongluo Decoction (YQTLT), Tongqiao Huoxue Tang (TQHXT), Tongluo Xifeng Decoction (TLXFT), Tongluo Fuzheng Decoction (TLFZT), Xuefu Zhuyu Decoction (XFZYT), Xiaoxuming Decoction (XXMT), Qufeng Xingxue Tongluo Formula (QFXXTLF), Banxia Baizhu Tianma Decoction (BXBZTMT), Buyang Huanwu Tang (BYHWT),Huatan Tongluo Decoction (HTTLT), Yiqi Huoxue Tongluo Decoction (YQHXTLT), Yiqi Huoxue Decoction (YQHXT), and Yiqi Huoxue Kaiqiao Prescription (YQHXKQP). Of them, XFZYT was most effective in reducing the NIHSS score; SQTLF was most effective in increasing the Barthel Index (BI) score; and HTTLT was most effective in improving activities of daily living (ADL).

**Conclusion:**

This network meta-analysis provided data on the relative efficacy of different TCM decoctions. Of them, XFZYT was most effective in reducing the NIHSS score; SQTLF was most effective in increasing the BI score; and HTTLT was most effective in improving the ADL score. At the same time, overall, XFZYT ranked first with its best efficacy regarding all the three outcome measures above, and SQTLF came second with its impact on two of the outcome measures.

## 1 Introduction

Stroke is divided into ischemic strokes (IS) and hemorrhagic stroke (HS), of which IS the most common type of stroke. IS a clinical syndrome caused by insufficient cerebral blood and oxygen supply due to cerebrovascular lesions, which results in ischemic and hypoxic necrosis of local brain tissues, followed by neurological impairment. Main clinical symptoms include hemiplegia, aphasia, coma, and movement disorders, with high rates of morbidity, disability, recurrence, and fatality (Xie et al., 2022; García-Pérez et al., 2021). According to epidemiological surveys, stroke is the second leading cause of death and the leading cause of acquired long-term disability worldwide (Herpich and Rincon, 2020). About 15 million people are diagnosed with stroke each year globally (Iadecola et al., 2020), and about 80% of them are IS patients (Herpich and Rincon, 2020). A combination of genetic and environmental factors contribute to the occurrence of IS, with well-defined risk factors including coronary heart disease, hypertension, diabetes mellitus, and hyperhomocysteinemia. The aim of current clinical treatment is to restore blood flow in the ischemic penumbra, restore blood circulation, reduce the extent of core infarcts, and ultimately restore neurological function. The drugs and treatments in modern medicine have limited effects (Xu et al., 2023). IS in the hyperacute phase is mainly treated with pharmacological thrombolysis or vascular intervention,but the therapeutic time window for thrombolysis after IS onset is very short. The National Institute of Neurological Disorders and Stroke (NINDS) demonstrated that intravenous thrombolysis (IVT) with recombinant tissue plasminogen activator (rt-PA) was effective in patients with acute IS (AIS) up to 3 h after onset, and the Golden Hour’period was usually within only 3–4.5 h. If post-stroke patients miss the Golden Hour of IVT or fail to seek proper drug therapy (Rønning et al., 2019), followed by pharmacological intervention and rehabilitation, there will be a significantly high incidence of post-stroke sequelae, such as hemiplegia, cognitive impairment, dysphagia, speech disorders, and a variety of psychological and physiological problems.

Modern research has found that TCM has good efficacy in treating IS (Wei et al., 2024). TCM can reduce inflammatory response, oxidative stress, and apoptosis, improve energy metabolism, protect cerebral nerves, improve brain injury after IS, and reduce sequelae. In addition, a combination of TCM with modern medicine can achieve the effect of “1 + 1 > 2”. For example, exogenous stem cell transplantation combined with TCM can better repair damaged nerves and promote the reconstruction of the cerebral neural structure and the generation of different neuronal cell lineages required for functional regeneration after cerebral ischemic injury (Gao et al., 2020; Zhou et al., 2018). Currently there is controversy about TCM regimens in the treatment of IS. There is a lack of direct comparison among different TCM decoctions, and there are a wide variety of TCM regimens. Therefore, this network meta-analysis was conducted to compare the efficacy of different TCM decoctions in the treatment of IS patients. Hopefully, this meta-analysis will provide a rationale for the selection of traditional Chinese medicines for IS treatment.

## 2 Methods

This study was conducted and reported in accordance with the Preferred Reporting Items for Systematic Reviews and Meta-Analyses (PRISMA) statement (Moher et al., 2009). The study protocol has been registered in the International Prospective Register of Systematic Reviews (PROSPERO): https://www.crd.york.ac.uk/PROSPERO/display_record.php?RecordID=571089, with a number of CRD42024571089.

### 2.1 Literature search

The databases, including Cochrane Library, PubMed, Embase, Web of Science, China National Knowledge Infrastructure (CNKI), Wanfang Data, and VIP, were searched using a computer to collect randomized controlled trials (RCTs) of TCM decoctions in the treatment of IS patients, with a search time frame until April 2024. Search was conducted using the following subject headings and free-text words: a combination of ischemic stroke, Tang (decoction) + San (powder) + Fang (formula) + Ji (dosage form), randomized controlled, random grouping or randomized, and NIHSS + BI + ADL. The search strategy is detailed in Supplementary Material 1. To find more eligible studies, we looked for relevant references from the included papers. There were no restrictions on language, year of publication or type of publication.

### 2.2 Inclusion and exclusion criteria

Inclusion criteria: Participants: patients with a clinical diagnosis of IS or stroke; intervention: decoctions based on modified TCM formulas, at least once a day; comparison: Standards of Care (SOC) for IS or SOC developed by hospitals based on expert consensuses or clinical guidelines; outcomes: the NIHSS score as the primary outcome measure, and the BI score and ADL score as secondary outcome measures; study design: all the included studies were randomized controlled trials (RCTs). Exclusion criteria: Duplicates, animal studies, case reports, conference abstracts, reviews, unavailable full texts, studies including participants with other organic diseases as comorbidities.

### 2.3 Data extraction

EndNote 21 was used to create a library of the articles obtained. Literature screening was completed independently by two investigators. Firstly, duplicate articles were excluded. Then, articles were initially screened by regarding their titles and abstracts thoroughly according to the inclusion and exclusion criteria. Next, the articles passing the initial screening were rescreened by reading their full texts according to the inclusion and exclusion criteria. If the results were disputed at any stage, a third investigator was also involved in the discussion to reach a consensus. Information extracted from the included studies included first author, year of publication, sample size, gender, mean age, interventions, and outcome measures.

### 2.4 Quality assessment

The latest recommendations in the Version 2 of the Cochrane risk-of-bias tool for randomized trials (RoB 2) (Higgins et al., 2019) were used to assess the risk of bias, including the following five main domains: bias arising from the randomization process, bias due to deviations from intended interventions, bias due to missing outcome data, bias in measurement of the outcome, and bias in selection of the reported result. In addition, the studies were rated as “low risk”, “unclear risk “or “high risk “of bias. Two assessors independently conducted the quality assessment, and cross-checked the results. Any discrepancies were resolved through discussion or consultation with a third investigator. Then, a schematic diagram of the results of risk of bias assessment was drawn by ReviewManager 5.3.

### 2.5 Data analysis

Bayesian network meta-analysis of data was performed using R4.3.1 (R Foundation for Statistical Computing) and STATA 15.0 (Stata Corp., College Station, TX, United States) to compare different interventions. A Markov Chain Monte Carlo (MCMC) method (Jansen et al., 2008) was used to obtain the best pooled estimates and probabilities for various treatment regimens, thereby assessing the relative efficacy and rank order of different treatment regimens. Continuous outcomes were expressed as the posterior mean difference (MD) along with its corresponding 95% confidence interval (CI). The probability of being the best intervention corresponding to an outcome measure for IS was predicted by calculating the surface under the cumulative ranking curve (SUCRA) value.

Network and funnel plots were drawn using STATA 15.0 to visualize direct and indirect comparative relationships among different treatment regimens, and to detect publication bias and other small-study effects. A metan command was installed to adjust the corresponding TCM decoctions. In a plot, each circle corresponds to a drug, and the edges represent existing comparisons. The size of each circle is proportional to the study size (number of patients included). A cumulative probability plot was drawn using the ggplot2 package.

## 3 Results

### 3.1 Process and results of literature screening

**FIGURE 1:**
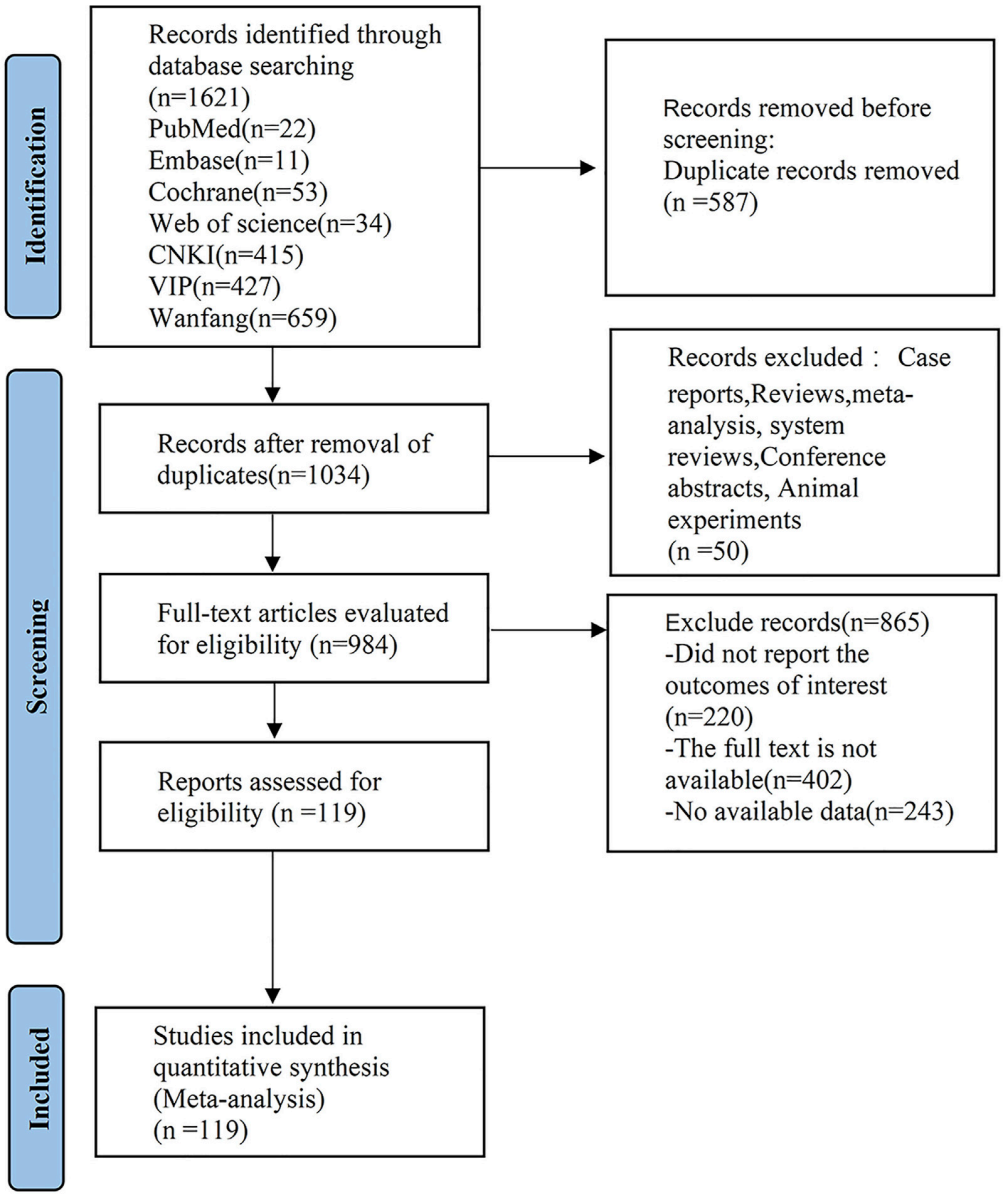
PRISlMA flow diagram of the study process. PRISMA, Preferred Reporting Items for Systematic review and Meta analysis.

A preliminary search in the databases yielded 1,621 articles. After the removal of 587 duplicates, 50 studies, including reviews, systematic reviews, and animal experiments, were excluded by reading their titles and abstracts. 622 articles were excluded by reading their full texts. 243 articles containing less than three relevant references were removed. In the end, 119 articles were included in the analysis (Figure 1).

### 3.2 General characteristics and risk of bias assessment of the included articles

The 119 articles included in the analysis involved 12,137 IS patients, and a total of 19 TCM decoctions including XLCQT, BXBZTMT, BYHWT, HTTLT, and XFZYT. The characteristics of the articles are detailed in Table 1. Of the included studies, some did not clearly state blinding methods, and two used non-randomized methods in the random sequence generation process and thus gave rise to a high risk of bias. Another high risk of bias arises mainly from incomplete data on outcome measures due to data loss. The risk of bias assessment of the included studies is shown in Table 2.

**TABLE 1:** Characteristics of the studies included in this network meta-analysis.

Study	Year	Sample size	Gender (M/F)	Mean age	Intervention	Outcome
L Zhang	2023	XLCQT:46 SOC:46	58/34	XLCQT:61.39 SOC:61.39	XLCQT: 400 mL Twice/day	NIHSS
J Ma	2020	XLCQT:48 SOC:48	53/43	XLCQT:62.12 SOC:62.25	XLCQT:300 mL Twice/day	NIHSS; BI; ADL
SH Yin	2018	XLCQT:41 SOC:39	51/29	XLCQT:64.51 SOC:65.08	XLCQT: Twice/day	NIHSS; BI
HW Lu	2020	XLCQT:37 SOC:37	41/33	XLCQT:65.49 SOC:65.32	XLCQT: Twice/day	NIHSS; BI
Q Zhao	2017	XLCQT:43 SOC:43	57/29	XLCQT:57.33 SOC:58.09	XLCQT: Twice/day	NIHSS
J Shi	2019	SQTLF:103 SOC:103	112/94	SQTLF:57.24 SOC:56.87	SQTLF:200 mL Twice/day	NIHSS
RR li	2022	SQTLF:60 SOC:60	79/41	SQTLF:60.32 SOC:60.28	SQTLF:200 mL Twice/day	NIHSS
H Han	2022	SQTLF:29 SOC:29	37/21	SQTLF:59.46 SOC:59.34	SQTLF:500 mL Twice/day	NIHSS; BI
HR Han	2021	ZFJXT:68 SOC:67	73/62	ZFJXT:68 SOC:67	ZFJXT: Once/day	NIHSS
XC Shen	2018	ZFJXT:60 SOC:60	75/45	ZFJXT:72.1 SOC:72.41	ZFJXT:300 mL Twice/day	NIHSS
XM Pan	2018	ZFJXT:40 SOC:40	41/39	ZFJXT:67.45 SOC:67.45	ZFJXT: Once/day	NIHSS
LN Zhang	2017	TQHXT:40 SOC:40	52/28	TQHXT:53.25 SOC:53.05	TQHXT:500 mL Twice/day	NIHSS; BI
Y Jiang	2016	TQHXT:40 SOC:40	52/28	TQHXT:53.25 SOC:53.05	TQHXT:200 mL Twice/day	NIHSS; BI
YJ Li	2022	TQHXT:45 SOC:45	57/33	TQHXT:65.03 SOC:64.97	TQHXT:300 mL Twice/day	NIHSS
SF Mao	2022	TQHXT + BAOOA:50 SOC:50	51/49	TQHXT:63.45 SOC:63.34	TQHXT:200 mL Twice/day	NIHSS; BI
X Liu	2022	TQHXT + TCMr:48 SOC:48	52/44	TQHXT:55.21 SOC:56.30	TQHXT: Twice/day	NIHSS
YL Shang	2022	TLXFT:45 SOC:45	53/37	TLXFT:58.03 SOC:57.62	TLXFT:300 mL Twice/day	NIHSS
XJ Chen	2018	TLXFT:90 SOC:90	102/78	TLXFT:58.9 SOC:58.2	TLXFT: Twice/day	ADL
RF Huang	2016	TLXFT:56 SOC:56	75/37	TLXFT:62.14 SOC:61.59	TLXFT: Twice/day	ADL
J Liang	2021	TLXFT:45 SOC:45	49/41	TLXFT:58.12 SOC:58.39	TLXFT: Twice/day	ADL
Y Cui	2022	TLFZT:44 SOC:44	57/31	TLFZT:64.15 SOC:64.29	TLFZT:400 mL Twice/day	NIHSS; ADL
J Song	2021	TLFZT:49 SOC:49	53/45	TLFZT:55.59 SOC:56.21	TLFZT:300 mL Twice/day	NIHSS; BI
GP Chen	2022	TLFZT:42 SOC:42	43/41	TLFZT:72.59 SOC:72.74	TLFZT: Twice/day	NIHSS; BI
J Ding	2020	XFZYT:63 SOC:63	77/49	XFZYT:63.2 SOC:63.6	XFZYT:150 mL Twice/day	NIHSS; BI
HL Liu	2018	XFZYT:50 SOC:50	58/42	XFZYT:62.11 SOC:63.02	XFZYT:300 mL Twice/day	NIHSS
HW Gao	2022	XFZYT:28 SOC:28	30/26	XFZYT:56.92 SOC:58.38	XFZYT:200 mL Twice/day	NIHSS; ADL
H Yang	2020	XFZYT:82 SOC:82	89/75	XFZYT:54.9 SOC:56.1	XFZYT: Twice/day	NIHSS
DJ Tang	2016	XFZYT:50 SOC:50	55/45	XFZYT + WM:60.34 SOC:60.14	XFZYT: Twice/day	NIHSS; ADL
KY Fang	2020	XFZYT:40 SOC:40	46/34	XFZYT:59.35 SOC:59.41	XFZYT: Twice/day	NIHSS
MH Du	2018	XFZYT:35 SOC:35	48/22	XFZYT:64.52 SOC:64.50	XFZYT:300 mL Twice/day	NIHSS
J Xu	2023	XFZYT:30 SOC:30	30/30	XFZYT:61.67 SOC:61.34	XFZYT: Once/day	NIHSS
H Gao	2017	XFZYT:40 SOC:40	48/32	XFZYT:68.93 SOC:69.21	XFZYT: Twice/day	NIHSS
J Liu	2017	XFZYT:53 SOC:53	59/47	XFZYT:61 SOC:63	XFZYT: Twice/day	NIHSS; BI
F Wang	2019	XFZYT:100 SOC:100	125/75	XFZYT:62.1 SOC:64.1	XFZYT: Twice/day	NIHSS
YH Li	2013	XFZYT:36 SOC:36	50/22	XFZYT:63.43 SOC:63.67	XFZYT:500 mL Twice/day	BI; NIHSS
DS Li	2020	XFZYT:63 SOC:63	68/58	XFZYT:62.74 SOC:63.25	XFZYT: Twice/day	NIHSS
WD Zhu	2015	XFZYT:80 SOC:80	108/52	XFZYT:70.11 SOC:73.31	XFZYT: Twice/day	NIHSS; BI
CM Zheng	2015	XFZYT:40 SOC:40	42/38	XFZYT:42.7 SOC:42.6	XFZYT: Tid	NIHSS
L Li	2020	XXMT:45 SOC:45	51/39	XXMT:65.54 SOC:64.98	XXMT:360 mL Twice/day	NIHSS; BI
ZC Jiang	2019	XXMT:31 SOC:31	33/29	XXMT:66.25 SOC:65.8	XXMT:200 mL Twice/day	NIHSS
SJ Ai	2022	XXMT:40 SOC:40	57/23	XXMT:66.63 SOC:66.41	XXMT:400 mL Twice/day	ADL
Q Zhang	2023	XXMT:31 SOC:31	35/27	XXMT:64.31 SOC:64.88	XXMT:300 mL Twice/day	NIHSS; ADL
XJ Liu	2018	XXMT:40 SOC:40	49/31	XXMT:60.01 SOC:59.26	XXMT:300 mL Twice/day	NIHSS
ZQ Sun	2017	QFHYXXTLF:42 SOC:42	51/33	QFHYXXTLF:59.79 SOC:60.17	QFHYXXTLF:400 mL Twice/day	NIHSS
RJ Zhu	2015	QFHYXXTLF:40 SOC:40	47/33	QFHYXXTLF:58.2 SOC:57.3	QFHYXXTLF:400 mL Twice/day	NIHSS
HM Shao	2015	QFHYXXTLF:40 SOC:40	47/33	QFHYXXTLF:58.2 SOC:57.3	QFHYXXTLF:400 mL Twice/day	NIHSS
ZH Zhang	2017	BXBZTMT:35 SOC:35	47/23	BXBZTMT:60.25 SOC:60.65	BXBZTMT: Twice/day	NIHSS; ADL
Q Zhou	2019	BXBZTMT:15 SOC:15	13/17	BXBZTMT:65.61 SOC:65.58	BXBZTMT:300 mL Twice/day	NIHSS
F Liu	2023	BXBZTMT:48 SOC:48	52/44	BXBZTMT:61.24 SOC:61.32	BXBZTMT:300 mL Twice/day	NIHSS
KL Fu	2018	BXBZTMT:73 SOC:73	81/65	BXBZTMT:59.93 SOC:59.38	BXBZTMT:300 mL Twice/day	NIHSS
Y Tang	2019	BXBZTMT:92 SOC:91	107/76	BXBZTMT:57.85 SOC:58.92	BXBZTMT:300 mL Twice/day	NIHSS
XQ Xun	2019	BXBZTMT:44 SOC:44	52/36	BXBZTMT:61.9 SOC:62.6	BXBZTMT:200 mL Twice/day	NIHSS; BI
F Liu	2022	BXBZTMT:40 SOC:40	52/28	BXBZTMT:58.19 SOC:57.20	BXBZTMT:400 mL Once/day	NIHSS
Q Tang	2017	BXBZTMT:40 SOC:40	42/38	BXBZTMT:64.0 SOC:63.1	BXBZTMT: Twice/day	NIHSS; ADL
JF Zou	2021	BXBZTMT:38 SOC:38	42/34	BXBZTMT:52.5 SOC:52.0	BXBZTMT: Twice/day	NIHSS
T Lei	2021	BXBZTMT:60 SOC:60	64/56	BXBZTMT:67.21 SOC:67.12	BXBZTMT:300 mL Twice/day	NIHSS; ADL
Q Zou	2021	BXBZTMT:43 SOC:43	49/37	BXBZTMT:58.4 SOC:58.6	BXBZTMT: Twice/day	NIHSS; BI
SL Liang	2023	BYHWT:59 SOC:58	65/52	BYHWT:68.3 SOC:66.8	BYHWT:200 mL Twice/day	NIHSS
GH Zhao	2014	BYHWT:34 SOC:34	33/35	BYHWT:63.6 SOC:63.6	BYHWT: Twice/day	NIHSS
L Lin	2022	BYHWT:42 SOC:42	47/37	BYHWT:58.4 SOC:56.3	BYHWT:300 mL Twice/day	NIHSS
GH Zhao	2014	BYHWT:32 SOC:32	33/31	BYHWT:62.5 SOC:66.9	BYHWT:300 mL Twice/day	NIHSS
GH Sun	2015	BYHWT:39 SOC:39	49/29	BYHWT:62.56 SOC:62.56	BYHWT: Twice/day	NIHSS
M Wu	2014	BYHWT:38 SOC:37	40/35	BYHWT:61.82 SOC:60.10	BYHWT: Twice/day	NIHSS; BI
Y Wang	2019	BYHWT:50 SOC:50	56/44	BYHWT:65.22 SOC:64.60	BYHWT:400 mL Twice/day	ADL; BI; NIHSS
H Xu	2017	BYHWT:85 SOC:85	92/78	BYHWT:65.33 SOC:65.16	BYHWT:100 mL Twice/day	ADL; NIHSS
XH Li	2024	BYHWT:52 SOC:52	65/39	BYHWT:68.04 SOC:67.90	BYHWT:300 mL Twice/day	NIHSS
AX Xu	2017	BYHWT:30 SOC:30	35/25	BYHWT:64.41 SOC:64.28	BYHWT: Twice/day	NIHSS; BI
LF Li	2022	BYHWT:30 SOC:30	33/27	BYHWT:63.9 SOC:63.5	BYHWT:300 mL Twice/day	NIHSS
PY Guo	2018	BYHWT:60 SOC:60	83/37	BYHWT:36.82 SOC:37.14	BYHWT:400 mL Twice/day	NIHSS
BJ Sheng	2023	BYHWT:48 SOC:48	60/36	BYHWT:60.11 SOC:58.92	BYHWT: Twice/day	NIHSS; BI
HT Mo	2016	BYHWT:43 SOC:43	45/41	BYHWT:61.25 SOC:61.30	BYHWT: Twice/day	NIHSS
WQ Chen	2021	BYHWT:46 SOC:46	60/32	BYHWT:42.98 SOC:41.53	BYHWT: Twice/day	NIHSS; BI
T Liu	2018	BYHWT:80 SOC:80	85/75	BYHWT:61.2 SOC:60.6	BYHWT:400 g Twice/day	NIHSS; ADL
ZY Shi	2018	BYHWT:90 SOC:90	95/85	BYHWT:61.57 SOC:60.76	BYHWT:400 mL Twice/day	ADL; NIHSS
C Zhang	2020	BYHWT:55 SOC:55	61/49	BYHWT:68.01 SOC:67.31	BYHWT:200 mL Twice/day	NIHSS
P Li	2020	BYHWT:40 SOC:40	45/35	BYHWT:56.48 SOC:56.57	BYHWT:150 mL Twice/day	NIHSS
TM Li	2018	BYHWT:34 SOC:35	41/28	BYHWT:69.67 SOC:69.22	BYHWT:400 mL Twice/day	NIHSS
QZ Lu	2018	BYHWT:39 SOC:39	45/33	BYHWT:58.97 SOC:59.67	BYHWT: Twice/day	NIHSS; ADL
Z Wang	2022	BYHWT:43 SOC:43	45/41	BYHWT:60.9 SOC:58.3	BYHWT: Twice/day	NIHSS
W Huang	2019	BYHWT:38 SOC:38	52/24	BYHWT:68.0 SOC:67.5	BYHWT: Twice/day	NIHSS; BI
F Ma	2018	BYHWT:63 SOC:63	75/51	BYHWT:67.86 SOC:68.37	BYHWT:400 mL Twice/day	NIHSS; ADL
FF Zhong	2021	BYHWT:360 SOC:360	388/332	BYHWT:67.86 SOC:68.38	BYHWT:400 mL Twice/day	NIHSS; ADL
HS Li	2016	BYHWT:43 SOC:42	48/37	BYHWT:55.17 SOC:53.96	BYHWT:400 mL Twice/day	NIHSS; ADL
ZY Xu	2020	BYHWT:53 SOC:53	79/27	BYHWT:64.15 SOC:63.98	BYHWT:300 mL Twice/day	NIHSS
CH Yin	2012	BYHWT:38 SOC:38	42/34	BYHWT:56.2 SOC:53.5	BYHWT: Twice/day	NIHSS; ADL
YH Li	2013	BYHWT:48 SOC:48	62/34	BYHWT:60.53 SOC:60.67	BYHWT:400 mL Twice/day	NIHSS; ADL
GP Zheng	2014	BYHWT:40 SOC:40	44/36	BYHWT:62.93 SOC:64.50	BYHWT:400 mL Twice/day	NIHSS
TH Zhang	2023	BYHWT:60 SOC:60	78/42	BYHWT:65.27 SOC:69.43	BYHWT:200 mL Twice/day	NIHSS
HS Huang	2022	BYHWT:50 SOC:50	55/45	BYHWT:58.01 SOC:58.37	BYHWT:200 mL Twice/day	NIHSS; BI
AL Chen	2018	BYHWT:100 SOC:100	121/79	BYHWT:67.55 SOC:66.74	BYHWT:400 mL Twice/day	NIHSS; BI
ZQ Huang	2022	BYHWT:40 SOC:40	44/36	BYHWT:63.37 SOC:63.27	BYHWT:400 mL Twice/day	NIHSS
TJ Liang	2012	BYHWT:46 SOC:46	56/36	BYHWT:62.1 SOC:59.6	BYHWT:250 mL Twice/day	BI
WC Wang	2020	BYHWT:32 SOC:32	41/23	BYHWT:59.5 SOC:59.0	BYHWT:400 mL Twice/day	NIHSS; ADL
ML Chen	2022	BYHWT:45 SOC:45	47/43	BYHWT:56.89 SOC:56.71	BYHWT:200 mL Twice/day	NIHSS
Q Liu	2023	BYHWT:31 SOC:30	36/26	BYHWT:62.63 SOC:62.35	BYHWT: Twice/day	NIHSS; ADL
JP Yang	2023	BYHWT:41 SOC:41	46/36	BYHWT:69.04 SOC:68.71	BYHWT: Twice/day	NIHSS; BI
XH Tan	2014	BYHWT:50 SOC:50	63/37	BYHWT:64.3 SOC:62.1	BYHWT: Twice/day	NIHSS
CF Guan	2008	BYHWT:44 SOC:43	48/39	BYHWT:62.17 SOC:62.89	BYHWT: Once/day	NIHSS
XY Zhong	2020	HTTLT:45 SOC:45	53/37	HTTLT:63.18 SOC:62.35	BYHWT:200 mL Twice/day	BI
F Tan	2013	HTTLT:33 SOC:32	37/28	HTTLT:56.3 SOC:58.7	HTTLT: Twice/day	BI
ZY Pan	2020	HTTLT:46 SOC:46	49/43	HTTLT:60.14 SOC:59.67	HTTLT:300 mL Twice/day	NIHSS; ADL
XQ Xu	2019	HTTLT:63 SOC:57	77/43	HTTLT:66.70 SOC:66.30	HTTLT:200 mL Twice/day	NIHSS
SY Li	2020	HTTLT:51 SOC:50	60/41	HTTLT:52.91 SOC:52.67	HTTLT:200 mL Twice/day	NIHSS
QY Zhou	2021	HTTLT:30 SOC:30	38/22	HTTLT:62.8 SOC:62.4	HTTLT:400 mL Twice/day	NIHSS; BI
QS Su	2018	HTTLT:40 SOC:40	49/31	HTTLT:65.12 SOC:64.58	HTTLT:100 mL Twice/day	NIHSS; BI
SN Zou	2019	HTTLT:31 SOC:31	33/29	HTTLT:66.59 SOC:65.96	HTTLT:200 mL Twice/day	BI
ZJ Zhuang	2022	HTTLT:30 SOC:30	36/24	HTTLT:64.10 SOC:67.10	HTTLT:400 mL, Twice/day	NIHSS; BI
YP Fu	2018	YQHXTLT:60 SOC:60	74/46	YQHXTLT:62.1 SOC:60.2	YQHXTLT:400 mL Twice/day	BI; NIHSS
CM Deng	2018	YQHXTLT:40 SOC:40	38/42	YQHXTLT:56.4 SOC:55.4	YQHXTLT: Twice/day	BI; NIHSS
XQ Lu	2019	YQHXTLT:48 SOC:48	49/47	YQHXTLT:62.34 SOC:63.12	YQHXTLT:400 mL Twice/day	BI
SP Zhou	2019	YQHXTLT:65 SOC:65	77/53	YQHXTLT:66.6 SOC:68.6	YQHXTLT: Twice/day	BI; NIHSS
H Li	2018	YQHXTLF:39 SOC:39	45/33	YQHXTLF:63.09 SOC:63.52	YQHXTLF: 500 mL Twice/day	NIHSS
XM Guo	2018	YQHXTLT:40 SOC:40	42/38	YQHXTLT:60.94 SOC:62.11	YQHXTLT: 200 mL Twice/day	ADL
CX Zhao	2022	YQHXT:52 SOC:52	61/43	YQHXT:62.50 SOC:63.30	YQHXT: 300 mL Twice/day	BI; NIHSS
HT Li	2021	YQHXT:40 SOC:40	47/33	YQHXT:64.18 SOC:63.72	YQHXT: Twice/day	BI; NIHSS
SG Sun	2015	YQHXT:45 SOC:40	49/36	YQHXT:63.1 SOC:62.5	YQHXT: 200 mL Twice/day	NIHSS
JF Xiao	2020	YQHXT:48 SOC:48	53/43	YQHXT:68.93 SOC:69.02	YQHXT: 150 mL Twice/day	NIHSS
ZH Guo	2018	YQHXT:49 SOC:49	57/41	YQHXT:58.84 SOC:59.02	YQHXT: 300 mL Twice/day	NIHSS; BI
F Yang	2020	YQHXT:51 SOC:51	52/50	YQHXT:64.29 SOC:64.35	YQHXT: 220 mL Twice/day	NIHSS; BI

M/F, Male/Female; BI, barthel index; NIHSS, National Institutes of Health stroke scale; ADL, activity of daily living scale; SOC, standard of care; XLCQT, xinglou chengqi decoction; SQTLF, shenqi tongluo decoction; ZFJXT, zhongfeng jiuxian decoction; TQHXT, tongqiao huoxue tang or tongqiao huoxue decoction; TLXFT, tongluo xifeng decoction; TLFZT, tongluo fuzheng decoction; XFZYT, xuefu zhuyu decoction; XXMT, xiaoxuming decoction; QFHYXXTLF, dispelling pathogenic wind and expelling blood stasis for promoting blood circulation and dredging collateral prescription or Qufeng Xingxue Tongluo Formula; BXBZTMT, banxia baizhu tianma decoction; BYHWT, buyang huanwu tang or buyang huanwu decoction; HTTLT, huatan tongluo decoction; YQHXTLT, yiqi huoxue tongluo decoction; YQHXT, yiqi huoxue decoction.

**TABLE 2:** Summary of the risk of bias.

Study	Generation of random sequences	Allocation concealment	Blinding	Blinding of outcome evaluators	Incomplete data	Selective reporting	Other bias
L Zhang 2023	Unclear risk	Unclear risk	Unclear risk	Unclear risk	Low risk	Low risk	Unclear risk
J Ma 2020	Low risk	Unclear risk	Unclear risk	Unclear risk	Low risk	Low risk	Unclear risk
Q Zhao 2017	Unclear risk	Unclear risk	Unclear risk	Unclear risk	Low risk	Low risk	Unclear risk
HW Lu 2020	Unclear risk	Unclear risk	Unclear risk	Unclear risk	Low risk	Low risk	Unclear risk
SH Yin 2018	Low risk	Unclear risk	Unclear risk	Unclear risk	High risk	Low risk	Unclear risk
J Shi 2019	Low risk	Unclear risk	Unclear risk	Unclear risk	Low risk	Low risk	Unclear risk
RR li 2022	Low risk	Unclear risk	Unclear risk	Unclear risk	Low risk	Low risk	Unclear risk
H Han 2022	Low risk	Unclear risk	Unclear risk	Unclear risk	Low risk	Low risk	Unclear risk
HR Han 2021	Low risk	Unclear risk	Unclear risk	Unclear risk	Low risk	Low risk	Unclear risk
XC Shen 2018	Unclear risk	Unclear risk	Unclear risk	Unclear risk	Low risk	Low risk	Unclear risk
XM Pan 2018	Unclear risk	Unclear risk	Unclear risk	Unclear risk	High risk	Low risk	Unclear risk
LN Zhang 2017	Unclear risk	Unclear risk	Unclear risk	Unclear risk	Low risk	Low risk	Unclear risk
Y Jiang 2016	Low risk	Unclear risk	Unclear risk	Unclear risk	Low risk	Low risk	Unclear risk
YJ Li 2022	Low risk	Unclear risk	Unclear risk	Unclear risk	Low risk	Low risk	Unclear risk
SF Mao 2022	Unclear risk	Unclear risk	Unclear risk	Unclear risk	Low risk	Low risk	Unclear risk
X Liu 2022	Low risk	Unclear risk	Unclear risk	Unclear risk	Low risk	Low risk	Unclear risk
YL Shang 2022	Low risk	Unclear risk	Unclear risk	Unclear risk	Low risk	Low risk	Unclear risk
XJ Chen 2018	Low risk	Unclear risk	Unclear risk	Unclear risk	Low risk	Low risk	Unclear risk
RF Huang 2016	Unclear risk	Unclear risk	Unclear risk	Unclear risk	Low risk	Low risk	Unclear risk
J Liang 2021	Unclear risk	Unclear risk	Unclear risk	Unclear risk	Low risk	Low risk	Unclear risk
Y Cui 2022	Low risk	Unclear risk	Unclear risk	Unclear risk	Low risk	Low risk	Unclear risk
J Song 2021	Unclear risk	Unclear risk	Unclear risk	Unclear risk	Unclear risk	Low risk	Unclear risk
GP Chen 2022	High risk	Unclear risk	Unclear risk	Unclear risk	Low risk	Low risk	Unclear risk
J Ding 2020	Unclear risk	Unclear risk	Low risk	Unclear risk	Low risk	Low risk	Unclear risk
HL Liu 2018	Low risk	Unclear risk	Unclear risk	Unclear risk	Low risk	Low risk	Unclear risk
HW Gao 2022	Low risk	Unclear risk	Unclear risk	Unclear risk	Low risk	Low risk	Unclear risk
H Yang 2020	Unclear risk	Unclear risk	Unclear risk	Unclear risk	Low risk	Low risk	Unclear risk
DJ Tang 2016	Low risk	Unclear risk	Unclear risk	Unclear risk	Low risk	Low risk	Unclear risk
KY Fang 2020	Low risk	Unclear risk	Unclear risk	Unclear risk	Low risk	Low risk	Unclear risk
MH Du 2018	Low risk	Unclear risk	Unclear risk	Unclear risk	Low risk	Low risk	Unclear risk
J Xu 2023	Low risk	Unclear risk	Unclear risk	Unclear risk	Low risk	Low risk	Unclear risk
H Gao 2017	Low risk	Unclear risk	Unclear risk	Unclear risk	Low risk	Low risk	Unclear risk
J Liu 2017	Low risk	Unclear risk	Unclear risk	Unclear risk	Low risk	Low risk	Unclear risk
F Wang 2019	Low risk	Unclear risk	Unclear risk	Unclear risk	Low risk	Low risk	Unclear risk
YH Li 2013	Low risk	Unclear risk	Unclear risk	Unclear risk	Low risk	Low risk	Unclear risk
DS Li 2020	Low risk	Unclear risk	Unclear risk	Unclear risk	Low risk	Low risk	Unclear risk
WD Zhu 2015	Low risk	Unclear risk	Unclear risk	Unclear risk	Low risk	Low risk	Unclear risk
CM Zheng 2015	Unclear risk	Unclear risk	Unclear risk	Unclear risk	Low risk	Low risk	Unclear risk
L Li 2020	Unclear risk	Unclear risk	Unclear risk	Unclear risk	Low risk	Low risk	Unclear risk
ZC Jiang 2019	Unclear risk	Unclear risk	Unclear risk	Unclear risk	Low risk	Low risk	Unclear risk
SJ Ai 2022	Low risk	Unclear risk	Unclear risk	Unclear risk	Low risk	Low risk	Unclear risk
Q Zhang 2023	Low risk	Unclear risk	Unclear risk	Unclear risk	Low risk	Low risk	Unclear risk
XJ Liu 2018	Unclear risk	Unclear risk	Unclear risk	Unclear risk	Low risk	Low risk	Unclear risk
ZQ Sun 2017	Low risk	Unclear risk	Unclear risk	Unclear risk	Low risk	Low risk	Unclear risk
RJ Zhu 2015	Unclear risk	Unclear risk	Unclear risk	Unclear risk	Low risk	Low risk	Unclear risk
HM Shao 2015	Unclear risk	Unclear risk	Unclear risk	Unclear risk	Low risk	Low risk	Unclear risk
ZH Zhang 2017	Low risk	Unclear risk	Unclear risk	Unclear risk	Low risk	Low risk	Unclear risk
Q Zhou 2019	Low risk	Unclear risk	Unclear risk	Unclear risk	Unclear risk	Low risk	Unclear risk
F Liu 2023	Low risk	Unclear risk	Unclear risk	Unclear risk	High risk	Low risk	Unclear risk
KL Fu 2018	Unclear risk	Unclear risk	Unclear risk	Unclear risk	Low risk	Low risk	Unclear risk
Y Tang 2019	Low risk	Unclear risk	Unclear risk	Unclear risk	Low risk	Low risk	Unclear risk
XQ Xun 2019	Unclear risk	Unclear risk	Unclear risk	Unclear risk	Low risk	Low risk	Unclear risk
F Liu 2022	Unclear risk	Unclear risk	Unclear risk	Unclear risk	Low risk	Low risk	Unclear risk
Q Tang 2017	Unclear risk	Unclear risk	Unclear risk	Unclear risk	Low risk	Low risk	Unclear risk
JF Zou 2021	Low risk	Unclear risk	Unclear risk	Unclear risk	Low risk	Low risk	Unclear risk
T Lei 2021	Unclear risk	Unclear risk	Unclear risk	Unclear risk	Low risk	Low risk	Unclear risk
Q Zou 2021	Low risk	Unclear risk	Unclear risk	Unclear risk	Low risk	Low risk	Unclear risk
SL Liang	Unclear risk	Unclear risk	Unclear risk	Unclear risk	Low risk	Low risk	Unclear risk
GH Zhao 2014	Unclear risk	Unclear risk	Unclear risk	Unclear risk	Low risk	Low risk	Unclear risk
L Lin 2022	Unclear risk	Unclear risk	Unclear risk	Unclear risk	Low risk	Low risk	Unclear risk
GH Zhao 2014a	Unclear risk	Unclear risk	Unclear risk	Unclear risk	Low risk	Low risk	Unclear risk
GH Sun 2015	Unclear risk	Unclear risk	Unclear risk	Unclear risk	Low risk	Low risk	Unclear risk
M Wu 2014	Unclear risk	Unclear risk	Unclear risk	Unclear risk	Low risk	Low risk	Unclear risk
Y Wang 2019	Low risk	Unclear risk	Unclear risk	Unclear risk	Low risk	Low risk	Unclear risk
H Xu 2017	Low risk	Unclear risk	Unclear risk	Unclear risk	Low risk	Low risk	Unclear risk
XH Li 2024	Unclear risk	Unclear risk	Unclear risk	Unclear risk	Low risk	Low risk	Unclear risk
AX Xu 2017	Low risk	Unclear risk	Unclear risk	Unclear risk	Low risk	Low risk	Unclear risk
LF Li 2022	Low risk	Unclear risk	Unclear risk	Unclear risk	Low risk	Low risk	Unclear risk
PY Guo 2018	Low risk	Unclear risk	Unclear risk	Unclear risk	Low risk	Low risk	Unclear risk
BJ Sheng 2023	Low risk	Unclear risk	Unclear risk	Unclear risk	Low risk	Low risk	Unclear risk
HT Mo 2016	Unclear risk	Unclear risk	Unclear risk	Unclear risk	Low risk	Low risk	Unclear risk
WQ Chen 2021	Unclear risk	Unclear risk	Unclear risk	Unclear risk	Low risk	Low risk	Unclear risk
T Liu 2018	Low risk	Unclear risk	Unclear risk	Unclear risk	Low risk	Low risk	Unclear risk
ZY Shi 2018	Low risk	Unclear risk	Unclear risk	Unclear risk	Low risk	Low risk	Unclear risk
C Zhang 2020	Low risk	Unclear risk	Unclear risk	Unclear risk	Low risk	Low risk	Unclear risk
P Li 2020	Low risk	Unclear risk	Unclear risk	Unclear risk	Low risk	Low risk	Unclear risk
TM Li 2018	Low risk	Unclear risk	Unclear risk	Unclear risk	Low risk	Low risk	Unclear risk
QZ Lu 2018	Low risk	Unclear risk	Unclear risk	Unclear risk	Low risk	Low risk	Unclear risk
Z Wang 2022	Unclear risk	Unclear risk	Unclear risk	Unclear risk	Low risk	Low risk	Unclear risk
W Huang 2019	Low risk	Unclear risk	Unclear risk	Unclear risk	Low risk	Low risk	Unclear risk
F Ma 2018	Low risk	Unclear risk	Unclear risk	Unclear risk	Low risk	Low risk	Unclear risk
FF Zhong 2021	Unclear risk	Unclear risk	Unclear risk	Unclear risk	Low risk	Low risk	Unclear risk
HS Li 2016	Low risk	Unclear risk	Unclear risk	Unclear risk	Low risk	Low risk	Unclear risk
ZY Xu 2020	Low risk	Unclear risk	Unclear risk	Unclear risk	Low risk	Low risk	Unclear risk
CH Yin 2012	Unclear risk	Unclear risk	Unclear risk	Unclear risk	Low risk	Low risk	Unclear risk
YH Li 2013a	Unclear risk	Unclear risk	Unclear risk	Unclear risk	Low risk	Low risk	Unclear risk
GP Zheng 2014	Unclear risk	Unclear risk	Unclear risk	Unclear risk	Low risk	Low risk	Unclear risk
TH Zhang 2023	Unclear risk	Unclear risk	Low risk	Unclear risk	Low risk	Low risk	Unclear risk
HS Huang 2022	Low risk	Unclear risk	Unclear risk	Unclear risk	Low risk	Low risk	Unclear risk
AL Chen 2018	Unclear risk	Unclear risk	Unclear risk	Unclear risk	Low risk	Low risk	Unclear risk
ZQ Huang 2022	Low risk	Unclear risk	Unclear risk	Unclear risk	Low risk	Low risk	Unclear risk
TJ Liang 2012	Low risk	Unclear risk	Unclear risk	Unclear risk	Low risk	Low risk	Unclear risk
WC Wang 2020	Unclear risk	Unclear risk	Unclear risk	Unclear risk	Low risk	Low risk	Unclear risk
ML Chen 2022	Low risk	Unclear risk	Unclear risk	Unclear risk	Low risk	Low risk	Unclear risk
Q Liu 2023	Unclear risk	Unclear risk	Low risk	Unclear risk	Low risk	Low risk	Unclear risk
JP Yang 2023	Unclear risk	Unclear risk	Unclear risk	Unclear risk	Low risk	Low risk	Unclear risk
XH Tan 2014	Unclear risk	Unclear risk	Unclear risk	Unclear risk	Low risk	Low risk	Unclear risk
CF Guan 2008	Low risk	Unclear risk	Unclear risk	Unclear risk	Low risk	Low risk	Unclear risk
XY Zhong 2020	Unclear risk	Unclear risk	Unclear risk	Unclear risk	Low risk	Low risk	Unclear risk
F Tan 2013	Unclear risk	Unclear risk	Unclear risk	Unclear risk	Low risk	Low risk	Unclear risk
ZY Pan 2020	Unclear risk	Unclear risk	Unclear risk	Unclear risk	Low risk	Low risk	Unclear risk
XQ Xu 2019	Unclear risk	Unclear risk	Unclear risk	Unclear risk	Low risk	Low risk	Unclear risk
SY Li 2020	Low risk	Unclear risk	Unclear risk	Unclear risk	Low risk	Low risk	Unclear risk
QY Zhou 2021	Low risk	Unclear risk	Unclear risk	Unclear risk	Low risk	Low risk	Unclear risk
QS Su 2018	Low risk	Unclear risk	Unclear risk	Unclear risk	Low risk	Low risk	Unclear risk
SN Zou 2019	Unclear risk	Unclear risk	Unclear risk	Unclear risk	Low risk	Low risk	Unclear risk
ZJ Zhuang 2022	Low risk	Unclear risk	Unclear risk	Unclear risk	Low risk	Low risk	Unclear risk
YP Fu 2018	Unclear risk	Unclear risk	Unclear risk	Unclear risk	High risk	Low risk	Unclear risk
CM Deng 2018	Unclear risk	Unclear risk	Unclear risk	Unclear risk	Low risk	Low risk	Unclear risk
XQ Lu 2019	Low risk	Unclear risk	Unclear risk	Unclear risk	Low risk	Low risk	Unclear risk
SP Zhou 2019	Low risk	Unclear risk	Unclear risk	Unclear risk	Low risk	Low risk	Unclear risk
H Li 2018	Unclear risk	Unclear risk	Unclear risk	Unclear risk	Low risk	Low risk	Unclear risk
XM Guo 2018	Unclear risk	Unclear risk	Unclear risk	Unclear risk	Low risk	Low risk	Unclear risk
CX Zhao 2022	Low risk	Unclear risk	Unclear risk	Unclear risk	Low risk	Low risk	Unclear risk
HT Li 2021	Low risk	Unclear risk	Unclear risk	Unclear risk	Low risk	Low risk	Unclear risk
SG Sun 2015	High risk	Unclear risk	Unclear risk	Unclear risk	Low risk	Low risk	Unclear risk
JF Xiao 2020	Low risk	Unclear risk	Unclear risk	Unclear risk	Low risk	Low risk	Unclear risk
ZH Guo 2018	Low risk	Unclear risk	Unclear risk	Unclear risk	Low risk	Low risk	Unclear risk
F Yang 2020	Unclear risk	Unclear risk	Unclear risk	Unclear risk	Low risk	Low risk	Unclear risk

## 4 Results of the network meta-analysis

### 4.1 NIHSS score

**FIGURE 2:**
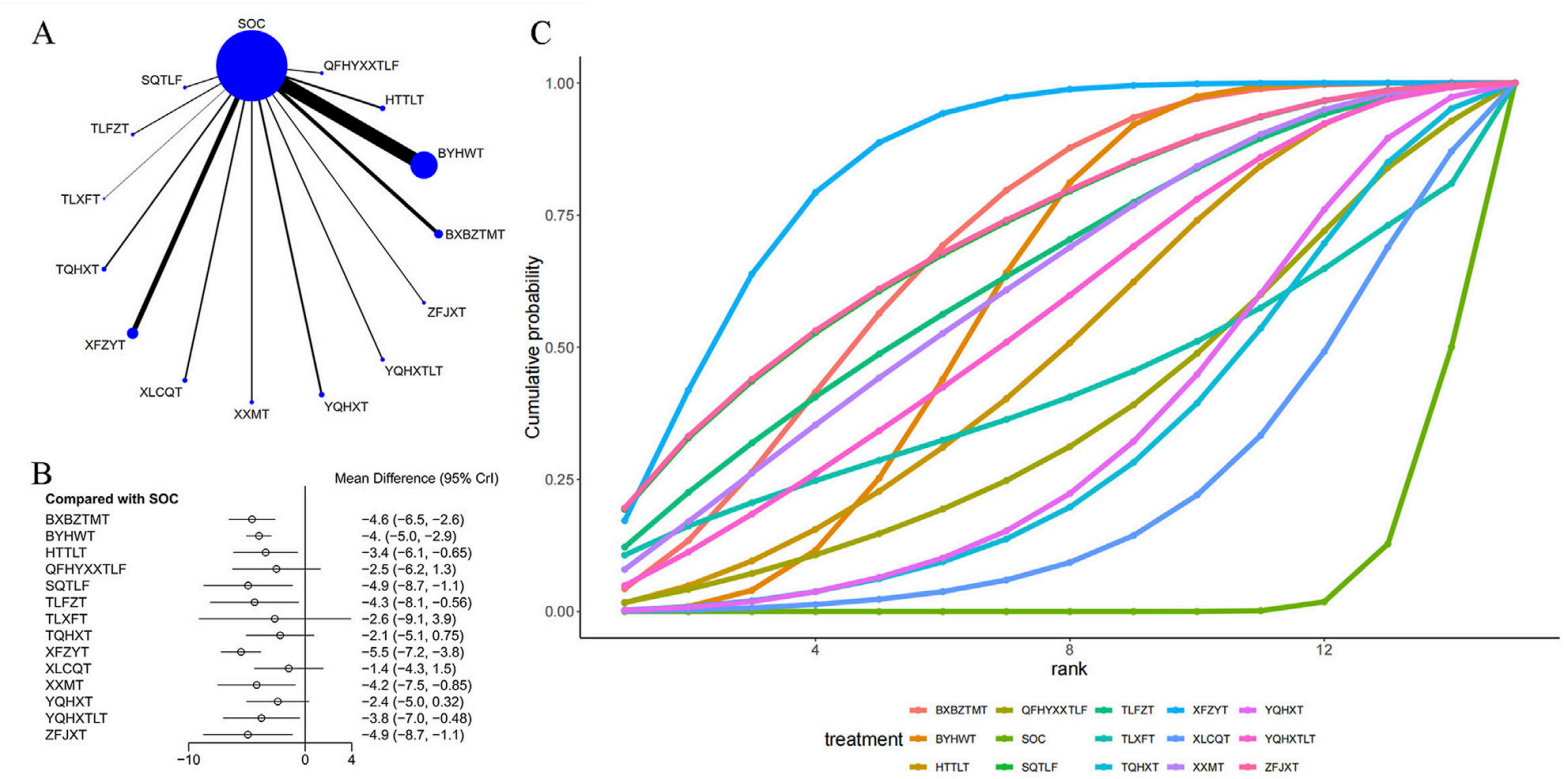
Effect of traditional Chinese medicine decoctions on NIHSS effect. **(A)** Network plot of comparisons for efficacy Nihss effects; **(B)** Forest plot of Nihss effect: Chinese medicine decoction vs. Standard of care; **(C)** Surface under the cumulative ranking curve plots for different Chinese medicine decoctions effects. The vertical axis represents cumulative probabilities and the horizontal axis represents rank. BXBZTMT, Banxia Baizhu Tianma decoction; BYHWT, Buyang Huanwu Tang or Buyang Huanwu decoction; HTTLT, Huatan Tongluo decoction; QFHYXXTLF, dispelling pathogenic wind and expelling blood stasis for promoting blood circulation and dredging collateral prescription or Qufeng Xingxue Tongluo Formula; SOC, Standard of care; SQTLF, Shenqi Tongluo decoction; TLFZT, Tongluo Fuzheng decoction; TLXFT, Tongluo Xifeng decoction; TQHXT, Tongqiao Huoxue Tang or Tongqiao Huoxue decoction; XFZYT, Xuefu Zhuyu decoction; XLCQT, Xinglou Chengqi decoction; XXMT, Xiaoxuming decoction; YQHXT, Yiqi Huoxue decoction; YQHXTLT, Yiqi Huoxue Tongluo decoction; ZFJXT, Zhongfeng Jiuxian decoction.

A total of 109 articles reported the NIHSS score, as shown in Table 1 and Supplementary Material 2. The network plot (Figures 2A) showed that a closed loop was not formed, and no interconnections were formed among different TCM decoctions. The included studies mostly investigated BYHWT, followed by XFZYT and HTTLT. As shown in Figures 2B, compared with SOC, BXBZTMT [MD = −4.6, 95% CI (−6.5, −2.6)], BYHWT [MD = −4., 95% CI (-5.0, −2.9)], HTTLT [MD = −3.4, 95% CI (−6.1, −0.65)], SQTLF [MD = −4.9, 95% CI (−8.7, −1.1)], TLFZT [MD = −4.3, 95% CI (−8.1, −0.56)], XFZYT [MD = −5.5, 95% CI (−7.2, −3.8)], XXMT [MD = −4.2, 95% CI (−7.5, −0.85)], YQHXTLT [MD = −3.8, 95% CI (−7.0, −0.48)], and ZFJXT [MD = −4.9, 95% CI (−8.7, −1.1)], were able to improve neurological deficits and reduce NIHSS scores in IS patients. SOC was mostly less effective than TCM decoctions. Of them, XFZYT was the most effective (−4.1 (−7.5, −0.7)). The results are shown in Supplementary Table S1 in Supplementary Material 3. The ranking of SUCRA values was as follows: XFZYT (84.3%) > ZFJXT (71.1%) > SQTLF (70.9%) > SOC(5%) (Figures 2C; Table 3).

**TABLE 3:** SUCRA of different Chinese medicine decoctions for various outcomes.

Treatment	NIHSS(%)	BI(%)	ADL (%)
BXBZTMT	69.1	77	41.1
BYHWT	58.6	50.1	46.2
HTTLT	49	55.6	94.8
QFHYXXTLF	36.5		
SOC	4.6	2.2	5.3
SQTLF	70.9	89.5	
TLFZT	63.4	41.9	57.6
TLXFT	41.6		66.4
TQHXT	30.5	15.9	
XFZYT	84.3	85.3	72.4
XLCQT	21.3	23.7	30.7
XXMT	61.2	29.3	36.9
YQHXT	32.9	44	
YQHXTLT	55	85.9	48.6
ZFJXT	71.1		

The redder the data, the higher the ranking of the drug in the outcome indicator.

### 4.2 BI score

**FIGURE 3:**
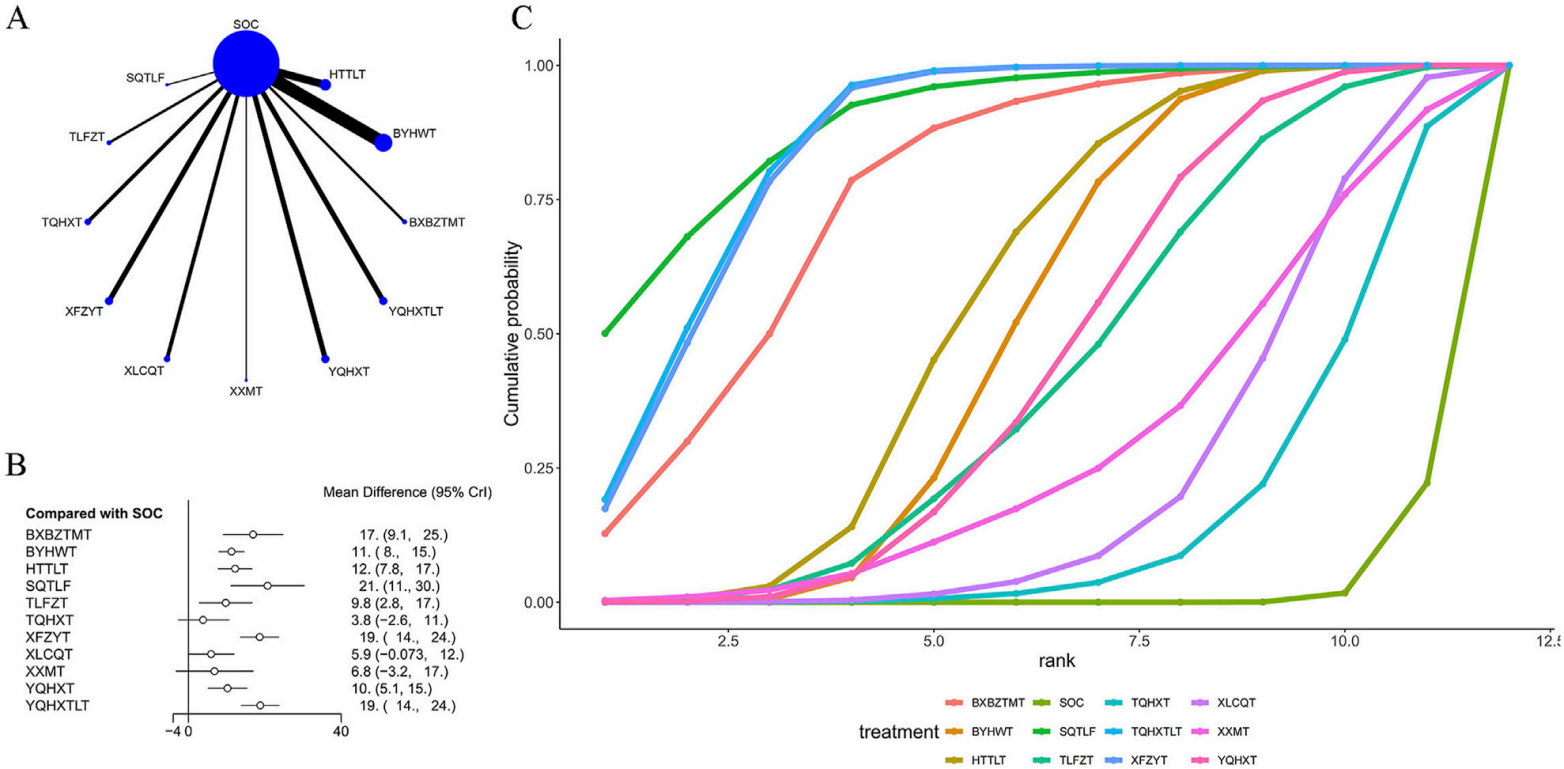
Effect of traditional Chinese medicine decoctions on BI effect. **(A)** Network plot of comparisons for efficacy BI effects; **(B)** Forest plot of BI effect: Chinese medicine decoction vs. Standard of care; **(C)** Surface under the cumulative ranking curve plots for different Chinese medicine decoctions effects. The vertical axis represents cumulative probabilities and the horizontal axis represents rank. BXBZTMT, Banxia Baizhu Tianma decoction; BYHWT, Buyang Huanwu Tang or Buyang Huanwu decoction; HTTLT, Huatan Tongluo decoction; SOC, Standard of care; SQTLF, Shenqi Tongluo decoction; TLFZT, Tongluo Fuzheng decoction; TQHXT, Tongqiao Huoxue Tang or Tongqiao Huoxue decoction; XFZYT, Xuefu Zhuyu decoction; XLCQT, Xinglou Chengqi decoction; XXMT, Xiaoxuming decoction; YQHXT, Yiqi Huoxue decoction; YQHXTLT, Yiqi Huoxue Tongluo decoction.

40 articles mentioned the BI score, as shown in Table 1. The network plot (Figures 3A) showed that no closed loop was formed, and no interconnections were formed among different TCM decoctions. The included studies mostly investigated BYHWT and HTTLT, and rarely investigated XXMT and SQTLF. As shown in Figures 3B, compared with SOC, BXBZTMT [MD = 17., 95% CI (9.1, 25.)], BYHWT [MD = 11., 95% CI (8., 15.)], HTTLT [MD = 12., 95% CI (7.8, 17.)], SQTLF [MD = 21., 95% CI (11., 30.)], TLFZT [MD = 9.8, 95% CI (2.8, 17.)], XFZYT [MD = 19., 95% CI (14., 24.)], YQHXT [MD = 10., 95% CI (5.1, 15.)], and YQHXTLT [MD = 19., 95% CI (14., 24.)] were able to enhance independence in patients. There was a significant difference between SOC and most TCM decoctions. Of TCM decoctions, SQTLF was most effective (−20.71 (−30.33, −11.13)). YQHXTLT and XFZYT were more effective than most other TCM decoctions, but their difference was not significant. The results are shown in Supplementary Table S2 in Supplementary Material 3. The ranking of SUCRA values was as follows: SQTLF (89.5%) > YQHXTLT (85.9%) > XFZYT (85.3%) > SOC (2.2%) (Figures 3C; Table 3).

### 4.3 ADL score

**FIGURE 4:**
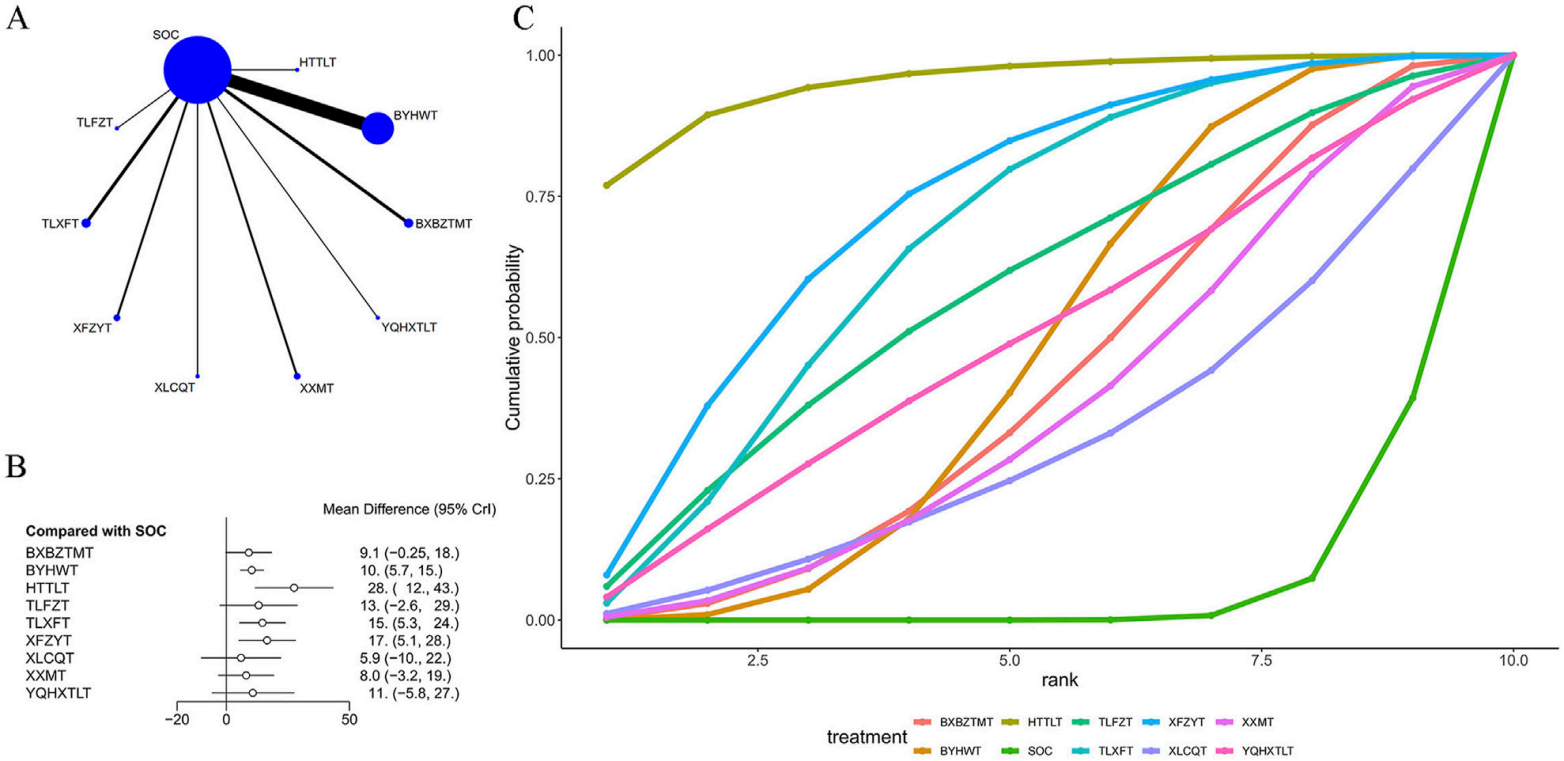
Effect of traditional Chinese medicine decoctions on ADL effect. **(A)** Network plot of comparisons for efficacy Nihss effects; **(B)** Forest plot of Nihss effect: Chinese medicine decoction vs. Standard of care; **(C)** Surface under the cumulative ranking curve plots for different Chinese medicine decoctions effects. The vertical axis represents cumulative probabilities and the horizontal axis represents rank. BXBZTMT, Banxia Baizhu Tianma decoction; BYHWT, Buyang Huanwu Tang or Buyang Huanwu decoction; HTTLT, Huatan Tongluo decoction; SOC, Standard of care; TLFZT, Tongluo Fuzheng decoction; TLXFT, Tongluo Xifeng decoction; XFZYT, Xuefu Zhuyu decoction; XLCQT, Xinglou Chengqi decoction; XXMT, Xiaoxuming decoction; YQHXTLT, Yiqi Huoxue Tongluo decoction.

26 articles used the ADL score as an outcome measure, as shown in Table 1. The network plot (Figures 4A) showed that no closed loop was formed, and no interconnections were formed among different TCM decoctions. The included studies mostly investigated BYHWT, TLXFT, and BXBZTMT, and rarely investigated other TCM decoctions. As shown in Figures 4B, compared with SOC, BYHWT [MD = 10., 95% CI (5.7, 15.)], HTTLT [MD = 28., 95% CI (12., 43.)], TLXFT [MD = 15., 95% CI (5.3, 24.)], and XFZYT [MD = 17., 95% CI (5.1, 28.)] were able to regulate functional status in individual patients to enable them to better perform various activities independently in daily life. SOC was found to be less effective than TCM decoctions. Of them, BXBZTMT (−18.38 (−36.83, −0.15)), BYHWT (−17.22 (−33.58, −0.59)), and XXMT (19.51 (0.06, 38.86)) were significantly different from HTTLT, but none of them was as effective as HTTLT, and HTTLT (27.5 (11.72, 43.36)) itself was most effective in improving the outcome measure. The results are shown in Supplementary Table S3 in Supplementary Material 3. The ranking of SUCRA values was as follows: HTTLT (94.8%) > XFZYT (72.4%) > TLXFT (66.4%) > SOC (5.3%) (Figures 4C; Table 3).

### 4.4 Pairwise comparisons

Regarding the NIHSS score, SOC was significantly different from BXBZTMT, BYHWT, HTTLT, SQTLF, TLFZT, TQHXT, XFZYT, XLCQT, XXMT, YQHXT, YQHXTLT, and ZFJXT. Regarding the BI score, SOC was significantly different from BXBZTMT, BYHWT, HTTLT, SQTLF, TLFZT, XFZYT, YQHXT, and YQHXTLT. Regarding the ADL score, SOC was significantly different from BYHWT, HTTLT, TLXFT, and XFZYT. The results corresponding to the three outcome measures are shown in Supplementary Material 4.

### 4.5 Publication bias

Regarding the outcome measures, their publication bias was assessed using funnel plots. The results are shown in Supplementary Figures S1–S3 in Supplementary Material 3. TCM decoctions were distinguished by different colors. As shown in the funnel plot of the NIHSS score (Supplementary Figure S1), the left and right sides were not completely symmetrical, with most of the data concentrated in the upper part, indicating that there was some publication bias. The funnel plot of the BI score (Supplementary Figure S2) was basically symmetrical, indicating less publication bias. The funnel plot of ADL (Supplementary Figure S3) was not completely symmetrical, indicating that there was some publication bias.

## 5 Discussion

An innovative aspect of this study is that we are the first to use a network meta-analysis to evaluate the efficacy of different TCM decoctions in IS patients.

This study found that regarding the therapeutic impact on the NIHSS score, XFZYT did the best, ZFJXT came second, while SQTLF came third and got close to ZFJXT. NIHSS score was used to systematically review the severity of neurological deficits in stroke patients. This study showed that XFZYT had a significant effect on all outcome measures, especially the NIHSS score. We therefore believed that overall, XFZYT had the best efficacy against IS. XFZYT originates from Yilin Gaicuo (Correction on Errors in Medical Works) written by Wang Qingren in Qing Dynasty. It is composed of 11 medicinal materials that promote blood circulation to remove blood stasis and relieve pain: Carthamus tinctorius, Semen persicae (peach kernels), Rehmannia glutinosa, Achyranthes root, Fructus aurantii, Bupleurum chinense, Paeonia veitchii, Platycodon grandiflorus, Glycyrrhizae Radix et Rhizoma (licorice), and Chuan Xiong (Chuanxiong rhizome) (Fu et al., 2020). XFZYT significantly improved the NIHSS score. This effect may be associated with its ingredients such as Amygdalin, Paeoniflorin, and Ligustrazine. These ingredients have been shown to have significant anti-inflammatory effects in relevant studies (Feng F. et al., 2024; Xu et al., 2024). In a study in a mouse model of IS (Yanfang Guo et al., 2020), it was found that luteolin in XFZYT inhibited not only microglia and astrocyte activation but also the HIF-1α/NLRP3 signaling pathway, thereby attenuating apoptosis of apoptosis of nerve cells, inflammation, and the degree of oxidative stress, so as to relieve cognitive impairmen (Zhang et al., 2022; Feng et al., 2021). On the other hand, found that kaempferol in the decoction not only modulated the classical pro-inflammatory NF-kB signaling pathway to promote the expression of anti-apoptotic proteins, inhibited neuronal death induced by cerebral ischemia and glial cell activation, reduced the activation and number of neutrophils in peripheral blood and brains of the rats, and significantly suppressed the levels of oxidative stress, inflammation, and apoptosis, which in turn alleviated IS . In addition, the components in the formula, such as Huang Qi and Dang Gui, enhance the body’s immunity and promote the repair of nerve cells. They help improve the recovery of neurological function after stroke by repairing neurological impairment and improving the NIHSS score (Wang et al., 2020).

XFZYT has also been shown to improve hemorheology and reduce blood viscosity, thereby promoting blood circulation and oxygen supply to the damaged brain tissues (Chen and Sui, 2020). Gao et al., (2024) showed that related serum biomarker levels were significantly reduced in patients after treatment with XFZYT, suggesting that it has a positive effect on inflammatory response and neural repair after stroke. Lee et al. (2011) also revealed that XFZYT might play a neuroprotective role by inhibiting HIF-1 and TNF-α to enhance the neuroprotective effect of rt-PA, and inhibit inflammation and apoptosis, thus improving neurological impairment. XFZYT has also shown efficacy in the treatment of hyperlipidemia to reduce the risk of IS by regulating blood lipids (Lee et al., 2021; Wenkai Yu et al., 2024; Xiangjun Zhong et al., 2018). Several studies have demonstrated that it exhibits significant efficacy in regulating cholesterol, improving inflammation and lipid metabolism, protecting vascular endothelial function and promoting neovascularization, as well as increasing patients’ BI score and ADL score to improve motor function and quality of life (Fu et al., 2024).

Regarding the therapeutic impact on the BI score, SQTLF did the best, YQHXTLT came second, and XFZYT came third. However, there were no significant difference among these TCM formulas, as shown in a league table. BI is often used to measure the ability to perform activities of daily living in patients with stroke or physical dysfunction, and is also often used for rehabilitation assessments, clinical research, and other scenarios (Shah et al., 1989). A higher BI score means that the patient is less dependent and is able to perform most of the activities of daily living.SQTLF replenishes qi, warms meridians to remove stagnation/stasis, promotes blood circulation, and unblocks collaterals. It is composed of Huang Qi (milkvetch root), Danshen (red sage), Pinellia Rhizome, Fructus aurantii, Poria cocos, Chuan Xiong, San Qi (Panax notoginseng), Dang Gui (Angelica sinensis), leeches, stir-fried Glycyrrhizae Radix et Rhizoma, Di Long (earthworm), and Radix Aucklandiae. In the past, SQTLF was frequently used to treat conditions mainly characterized by blood stasis (Jian et al., 2020). We believed that was why it performed best in improving the BI score. Huang Qi is the principal component (sovereign ingredient) in the formula. It replenishes qi and elevates yang to help strengthen healthy qi and improve qi flow and blood circulation. Danshen and Chuan Xiong boost blood flow to remove blood stasis, and unblock collaterals to remove stagnation. They are able to promote blood circulation and reduce blood viscosity, thereby improving microcirculation and cerebral blood flow supply. In addition, San Qi in the formula fights platelet aggregation to help prevent thrombosis and further promote the recovery of neurological function (Gao et al., 2014; Cong, 2021). A study by Zhang Yingfeng, et al. demonstrated that a lyophilisate of Danshen and Chuan Xiong significantly improved the cerebral lipidomic profile in a rat model of middle cerebral artery occlusion by regulating lipid metabolism to improve IS (Zhang et al., 2019). Fu Xueqin, et al. demonstrated that Danshen plus Chuanxiong was able to exert an anti-apoptotic effect through the PI3K/AKT signaling pathway to ameliorate cerebral ischemia/reperfusion injury in rats (Fu et al., 2022). These mechanisms of action were further evidenced by the findings of Wen Yijun, et al. Their research found that after treatment in the observational group, activated partial thromboplastin time (APTT), prothrombin time (PT), and clotting time (TT) were all prolonged, and the level of fibrinogen (FIB) was reduced (Wen, 2024). We therefore concluded that SQTLF promoted improved coagulation function through antiplatelet agglutination to regulate blood circulation and hemorheology and then promoted the recovery of neurological function, while protecting nerve cells through regulation of lipid metabolism and antiapoptotic function, thus significantly improving the BI score in post-stroke hemiplegic patients. SQTLF is able to improve patients’ ability to walk and take care of themselves in daily life.

It also had a significant effect in on the NIHSS score. Modern pharmacological research has demonstrated that this may be due to ingredients such as Ginsenoside 4 and Astragalus polysaccharide (Liu et al., 2023; Shi and Ma, 2024), which have a stronger affinity for the corresponding receptors to regulate the release of neurotransmitters and fight against oxidative stress, thus protecting nerve cells and promoting the recovery of nerve function. Therefore, we believed that SQTLF protected nerve cells and promoted the recovery of nerve function mainly by enhancing neurotransmitter release and fighting against oxidative stress. At the same time, it attenuated apoptosis, improved blood flow and inhibited inflammatory response to improve the NIHSS score in IS patients.

In this study, we investigated the impact of different TCM decoctions on the ADL score in IS patients. We found that HTTLT, XFZYT, and TLXFT all showed positive efficacy in improving the ADL score. HTTLT exhibited the best effect on this outcome measure. It is mainly composed of Danshen, Rhizoma Gastrodiae, Pinellia Rhizome, Poria cocos, unprepared Atractylodes rhizome, Xiang Fu (Rhizoma Cyperi), Rhei Radix et Rhizoma prepared with alcohol, Arisaema cum Bile (Dan Nanxing in Chinese, DNX), Bambusae Concretio Silicea, and San Qi. HTTLT plays a main role in breaking up phlegm, activating meridians, and boosting blood flow to remove blood stasis. HTTLT increased the BI score possibly due to its function to improve the internal environment so as to reduce pathological factors, thereby improving nerve function and motor function (Zhao et al., 2022). Tang San, et al. (Tang et al., 2023) found that HTTLT significantly reduced the NIHSS score after cerebral ischemia/reperfusion, increased the expression of brain-derived neurotrophic factor (BDNF), and reduced the levels of neuron-specific enolase (NSE), high-sensitivity C-reactive protein (hs-CRP), and homocysteine (Hcy) levels in rats. In addition, Luo Fanghe, et al. found that HTTLT significantly improve the limb motor function of post-stroke hemiplegic patients who was recovering (Luo, 2024).

Pharmacological studies have demonstrated that Danshen in the formula is able to reduce the incidence of brain infarction and attenuate nerve injury caused by ischemia/reperfusion (Chen et al., 2024). Di Long is often used to treat joint inflammation. In this prescription, Di Long was shown to ameliorate inflammation by inhibiting the activation of the NF-κB signaling pathway and modulating the Th1/Th2 balance (Bao et al., 2022). Atractylodes rhizome has immunomodulatory and anti-inflammatory effects (Feng J. M. et al., 2024). We believed that Atractylodes rhizome as an important ingredient of HTTLT might reduce inflammatory response in brain tissue to protect neurons. HTTLT improved limb motor function to increase the ADL score through its role in reducing neurological impairment, modulating immune response and reducing inflammation. Its value for long-term rehabilitation can be further explored in future studies.

This study explored the differences in efficacy among different TCM decoctions, but found no significant differences between these top-ranked interventions in the league table, possibly due to the impact of dose selection and frequency of oral medication. Our conclusion needs to be justified by more studies to provide IS patients with a choice of treatment. However, our study has some limitations. Firstly, there is a wide range of traditional Chinese medicines. This study reviewed the data on TCM decoctions only, and did not include many other types of traditional Chinese medicines, such as “creams”, “pills”, “granules”, and “Chinese patent medicines”. Secondly, the included studies were different in quality. Some of them did not clearly state methods for random assignment, contained small sample sizes, and failed to implement blinding strictly, thus possibly affecting the reliability of the results. Thirdly, there was large heterogeneity among the included studies regarding the treatment modality for patient populations. Fourthly, the inability to establish criteria involving dosage and strength in a unified manner for interventions may limit direct and indirect comparisons of outcomes. Fifthly, regarding the selection of outcome measures, cognitive indicators such as MOCCA and MSSE may be added in the future for comprehensive evaluation. Finally, we cannot completely rule out the effect of unmeasured confounders.

Based on this study, we concluded that TCM decoctions were able to improve outcome measures in the patients. Of them, XFZYT was most effective in reducing the NIHSS score, SQTLF was most effective in increasing the BI score, and HTTLT was most effective in improving the ADL score. At the same time, overall assessment showed that XFZYT ranked first with its best efficacy regarding all the three outcome measures above, and SQTLF came second with its impact on two of the outcome measures.

## Data Availability

The original contributions presented in the study are included in the article/Supplementary Material, further inquiries can be directed to the corresponding author.
