# Supplementary material for: Efficacy of different traditional Chinese medicine decoctions in the treatment of ischemic stroke: a network meta-analysis
Source: Front Pharmacol. 2024 Nov 1;15:1486458. doi: 10.3389/fphar.2024.1486458 (PMC11565597; doi:10.3389/fphar.2024.1486458)
Supplement: Supplementary file 1 [file DataSheet4.docx]

**Supplementary Material 4** Pairwise comparison

Nihss pairwise comparison

| Comparison | Number of studies | Heterogeneity | MD (95%CI) |
| --- | --- | --- | --- |
| **SOC vs BXBZTMT** | 11 | 93.0% | 4.5605 (2.5953, 6.5268) |
| **SOC vs BYHWT** | 40 | 97.8% | 3.9640 (2.9286, 5.0218) |
| **SOC vs HTTLT** | 6 | 90.5% | 3.3923 (0.65755, 6.1721) |
| **SOC vs QFHYXXTLF** | 3 | 0.0% | 2.4741 (−1.2917, 6.2519) |
| **SQTLF vs SOC** | 3 | 94.8% | −4.8945 (−8.6991, −1.1014) |
| **TLFZT vs SOC** | 3 | 97.1% | −4.3494 (−8.1235, −0.57795) |
| **TLXFT vs SOC** | 1 | None | −2.6379 (−9.0799, 3.8176) |
| **TQHXT vs SOC** | 5 | 99.0% | −2.1578 (−5.0685, 0.74599) |
| **XFZYT vs SOC** | 15 | 99.6% | −5.5134 (−7.2131, −3.8153) |
| **XLCQT vs SOC** | 5 | 99.8% | −1.4042 (−4.3407, 1.5412) |
| **XXMT vs SOC** | 4 | 97.0% | −4.1668 (−7.5102, −0.82672) |
| **YQHXT vs SOC** | 6 | 84.4% | −2.3405 (−5.0116, 0.33459) |
| **YQHXTLT vs SOC** | 4 | 98.4% | −3.7493 (−7.0534, −0.47177) |
| **ZFJXT vs SOC** | 3 | 94.3% | −4.9297 (−8.7129, −1.0872) |

BI pairwise comparison

| Comparison | Number of studies | Heterogeneity | MD (95%CI) |
| --- | --- | --- | --- |
| **SOC vs BXBZTMT** | 2 | 0.0% | −16.919 (−24.785, −9.0812) |
| **SOC vs BYHWT** | 10 | 92.8% | −11.276 (−14.558, −7.9319) |
| **SOC vs HTTLT** | 6 | 42.8% | −12.192 (−16.643, −7.7160) |
| **SQTLF vs SOC** | 1 | None | 20.758 (11.235, 30.424) |
| **TLFZT vs SOC** | 2 | 0.0% | 9.7412 (2.7752, 16.698) |
| **TQHXT vs SOC** | 3 | 95.9% | 3.7901 (−2.6400, 10.606) |
| **XFZYT vs SOC** | 4 | 98.8% | 18.637 (13.595, 23.660) |
| **XLCQT vs SOC** | 3 | 0.0% | 5.9078 (−0.11986, 11.911) |
| **XXMT vs SOC** | 1 | None | 6.8375 (−3.1720, 16.798) |
| **YQHXT vs SOC** | 4 | 60.1% | 10.244 (5.1652, 15.401) |
| **YQHXTLT vs SOC** | 4 | 88.0% | 18.823 (13.780, 23.793) |

ADL pairwise comparison

| Comparison | Number of studies | Heterogeneity | MD (95%CI) |
| --- | --- | --- | --- |
| **SOC vs BXBZTMT** | 3 | 82.7% | −9.1222 (−18.289, 0.19771) |
| **SOC vs BYHWT** | 12 | 99.2% | −10.281 (−15.109, −5.6802) |
| **SOC vs HTTLT** | 1 | None | −27.503 (−43.250, −11.899) |
| **TLFZT vs SOC** | 1 | None | 13.086 (−2.5846, 28.530) |
| **TLXFT vs SOC** | 3 | 79.5% | 14.629 (5.4277, 23.853) |
| **XFZYT vs SOC** | 2 | 98.3% | 16.539 (5.1304, 28.088) |
| **XLCQT vs SOC** | 1 | None | 5.9489 (−10.153, 21.963) |
| **XXMT vs SOC** | 2 | 23.3% | 8.0268 (−3.1162, 19.267) |
| **YQHXTLT vs SOC** | 1 | None | 10.779 (−5.8528, 27.326) |
