# Supplementary material for: Efficacy of different traditional Chinese medicine decoctions in the treatment of ischemic stroke: a network meta-analysis
Source: Front Pharmacol. 2024 Nov 1;15:1486458. doi: 10.3389/fphar.2024.1486458 (PMC11565597; doi:10.3389/fphar.2024.1486458)
Supplement: Supplementary file 2 [file DataSheet3.doc]

**Supplementary Material 3**

Figure S1: Funnel Plots of NIHSS


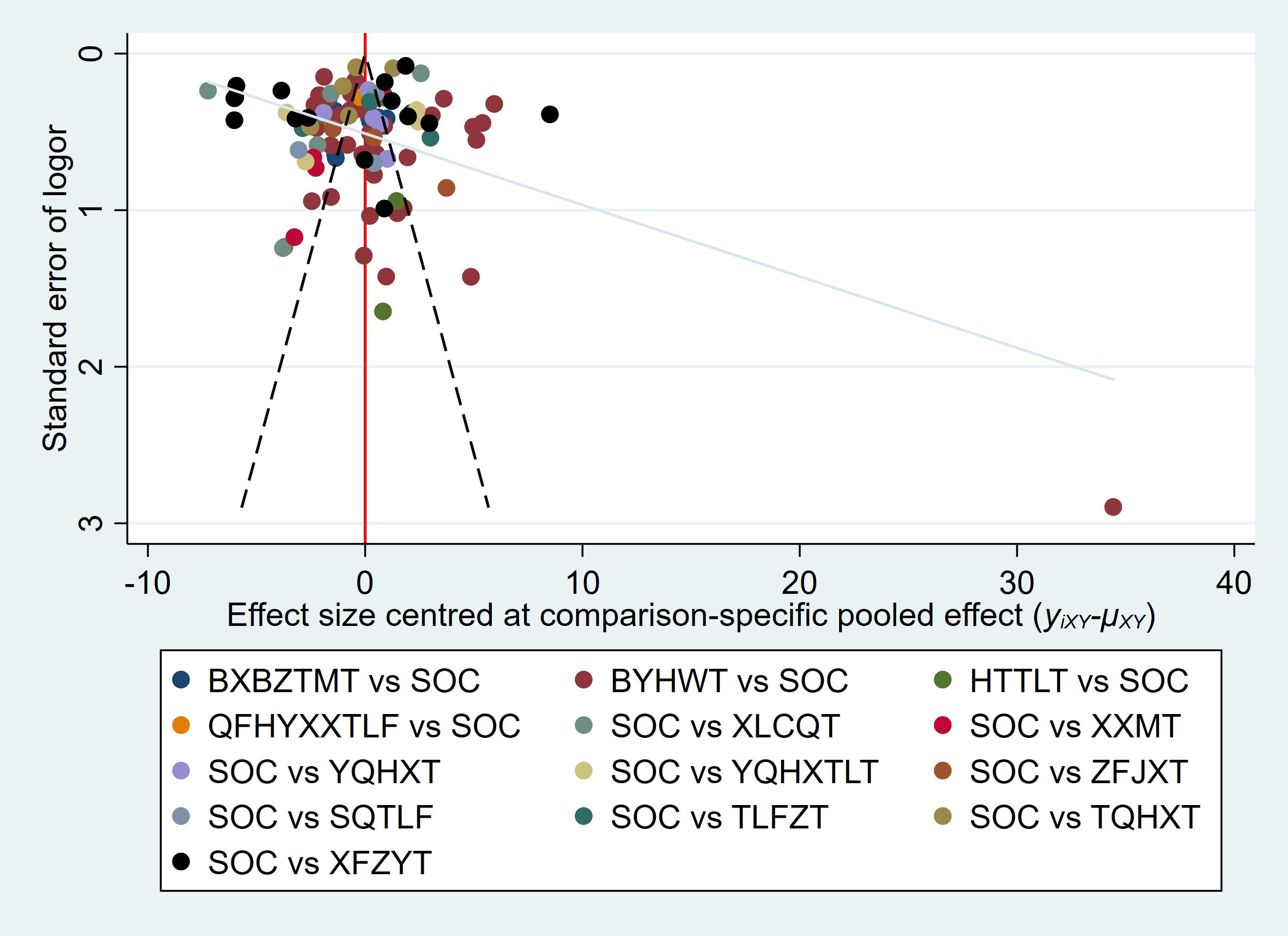


Figure S2: Funnel Plots of BI


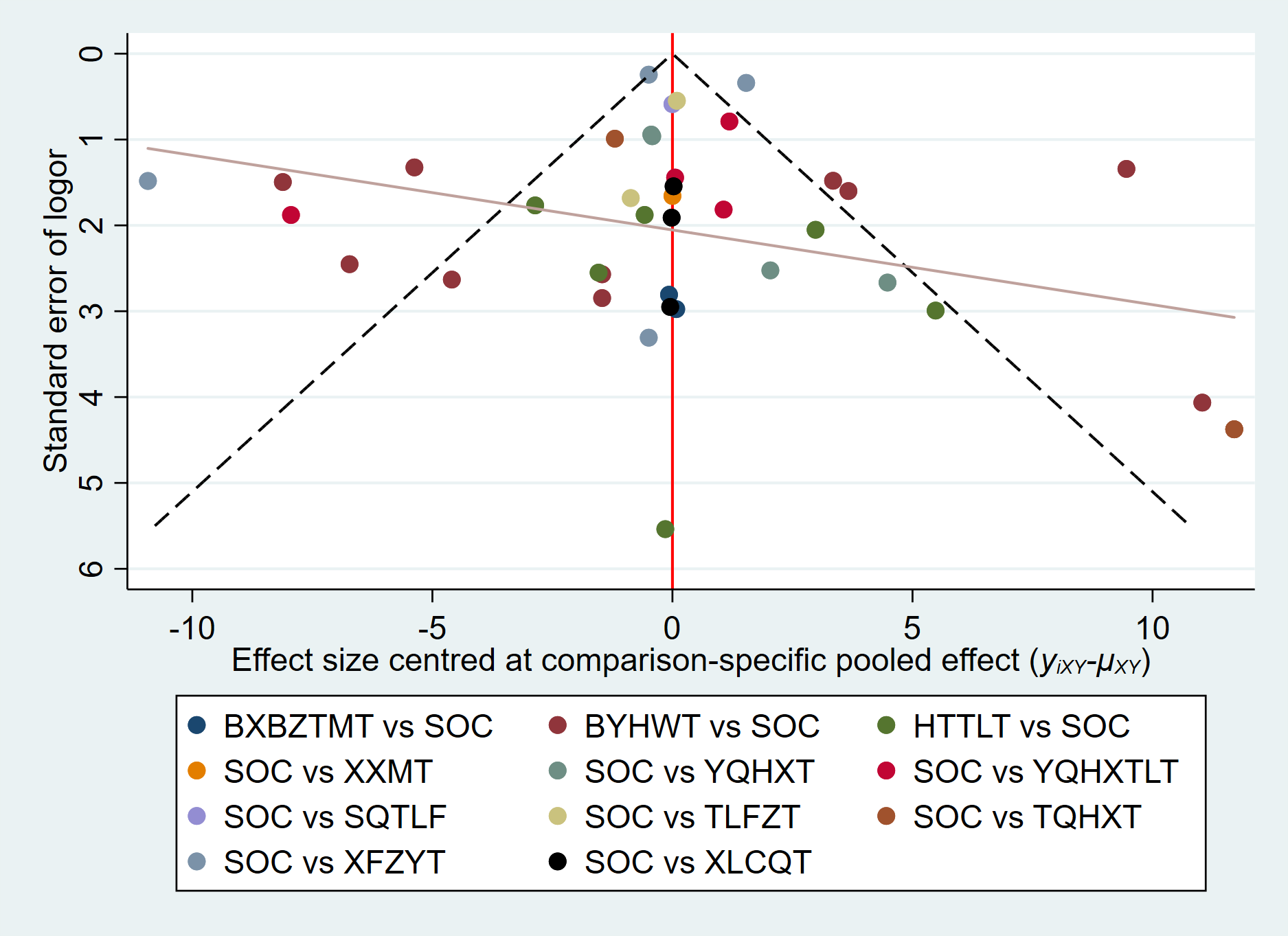


Figure S3: Funnel Plots of ADL


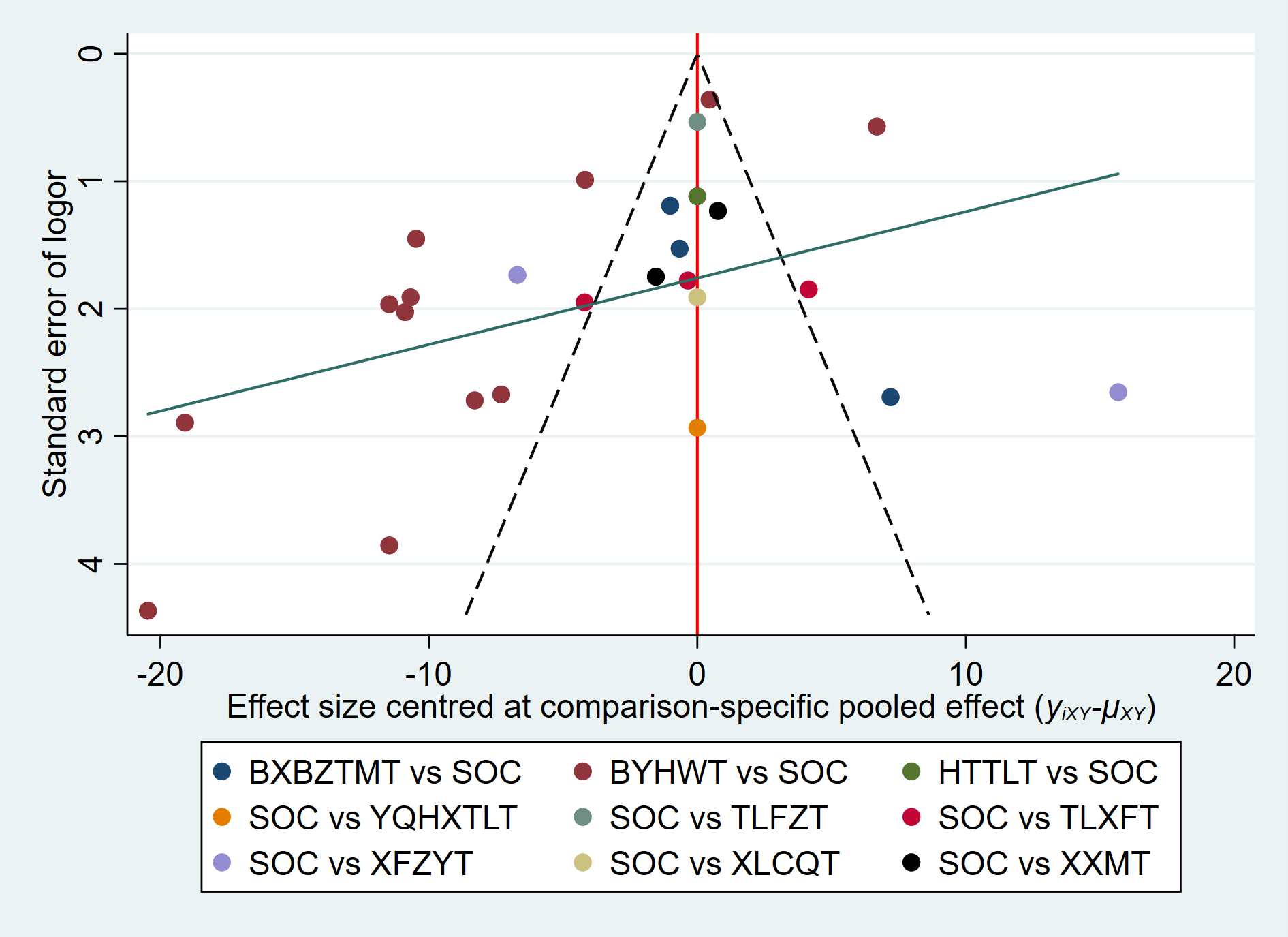


Table S1 Nihss League Table

| MD 95%CI | | | | | | | | | | | | | | |
| --- | --- | --- | --- | --- | --- | --- | --- | --- | --- | --- | --- | --- | --- | --- |
| BXBZTMT |  |  |  |  |  |  |  |  |  |  |  |  |  |  |
| -0.6 (-2.82, 1.66) | BYHWT |  |  |  |  |  |  |  |  |  |  |  |  |  |
| -1.17 (-4.56, 2.2) | -0.57 (-3.53, 2.36) | HTTLT |  |  |  |  |  |  |  |  |  |  |  |  |
| -2.1 (-6.35, 2.14) | -1.49 (-5.41, 2.4) | -0.93 (-5.58, 3.73) | QFHYXXTLF |  |  |  |  |  |  |  |  |  |  |  |
| -4.56 (-6.53, -2.59) | -3.96 (-5.03, -2.92) | -3.39 (-6.13, -0.65) | -2.47 (-6.22, 1.3) | SOC |  |  |  |  |  |  |  |  |  |  |
| 0.34 (-3.95, 4.61) | 0.94 (-3.01, 4.88) | 1.51 (-3.18, 6.2) | 2.44 (-2.92, 7.76) | 4.9 (1.09, 8.69) | SQTLF |  |  |  |  |  |  |  |  |  |
| -0.22 (-4.47, 4.05) | 0.38 (-3.55, 4.29) | 0.96 (-3.71, 5.61) | 1.88 (-3.46, 7.22) | 4.34 (0.56, 8.14) | -0.56 (-5.89, 4.82) | TLFZT |  |  |  |  |  |  |  |  |
| -1.95 (-8.76, 4.84) | -1.36 (-7.99, 5.21) | -0.78 (-7.84, 6.27) | 0.14 (-7.42, 7.64) | 2.61 (-3.91, 9.1) | -2.29 (-9.83, 5.23) | -1.73 (-9.29, 5.82) | TLXFT |  |  |  |  |  |  |  |
| -2.42 (-5.93, 1.09) | -1.82 (-4.91, 1.27) | -1.25 (-5.23, 2.75) | -0.33 (-5.06, 4.44) | 2.15 (-0.75, 5.06) | -2.75 (-7.51, 2.04) | -2.2 (-6.95, 2.54) | -0.46 (-7.56, 6.67) | TQHXT |  |  |  |  |  |  |
| 0.94 (-1.65, 3.54) | 1.54 (-0.46, 3.52) | 2.12 (-1.09, 5.34) | 3.04 (-1.09, 7.18) | 5.51 (3.82, 7.19) | 0.61 (-3.56, 4.77) | 1.17 (-2.98, 5.31) | 2.91 (-3.8, 9.65) | 3.36 (0, 6.71) | XFZYT |  |  |  |  |  |
| -3.15 (-6.7, 0.38) | -2.55 (-5.7, 0.56) | -1.98 (-6.03, 2.02) | -1.06 (-5.82, 3.71) | 1.41 (-1.54, 4.34) | -3.49 (-8.3, 1.3) | -2.94 (-7.74, 1.86) | -1.2 (-8.33, 5.95) | -0.74 (-4.88, 3.38) | -4.1 (-7.5, -0.7) | XLCQT |  |  |  |  |
| -0.39 (-4.27, 3.48) | 0.2 (-3.29, 3.68) | 0.78 (-3.56, 5.09) | 1.7 (-3.33, 6.73) | 4.17 (0.85, 7.49) | -0.73 (-5.78, 4.33) | -0.18 (-5.21, 4.85) | 1.56 (-5.74, 8.86) | 2.02 (-2.38, 6.44) | -1.34 (-5.07, 2.39) | 2.76 (-1.68, 7.2) | XXMT |  |  |  |
| -2.21 (-5.52, 1.11) | -1.61 (-4.48, 1.25) | -1.05 (-4.88, 2.8) | -0.11 (-4.73, 4.5) | 2.35 (-0.32, 5.02) | -2.55 (-7.19, 2.07) | -2 (-6.62, 2.63) | -0.26 (-7.28, 6.79) | 0.21 (-3.73, 4.14) | -3.16 (-6.33, 0) | 0.95 (-3.02, 4.9) | -1.82 (-6.07, 2.44) | YQHXT |  |  |
| -0.8 (-4.62, 3.02) | -0.21 (-3.66, 3.21) | 0.36 (-3.93, 4.64) | 1.29 (-3.71, 6.28) | 3.76 (0.48, 7.02) | -1.14 (-6.18, 3.88) | -0.59 (-5.59, 4.42) | 1.15 (-6.13, 8.43) | 1.61 (-2.77, 5.98) | -1.75 (-5.43, 1.92) | 2.35 (-2.04, 6.76) | -0.41 (-5.1, 4.25) | 1.4 (-2.83, 5.63) | YQHXTLT |  |
| 0.35 (-3.92, 4.63) | 0.95 (-3.01, 4.89) | 1.53 (-3.17, 6.2) | 2.45 (-2.91, 7.8) | 4.92 (1.1, 8.72) | 0.02 (-5.35, 5.39) | 0.58 (-4.79, 5.93) | 2.31 (-5.25, 9.82) | 2.77 (-2.03, 7.54) | -0.59 (-4.75, 3.55) | 3.51 (-1.3, 8.32) | 0.75 (-4.32, 5.82) | 2.57 (-2.1, 7.19) | 1.16 (-3.89, 6.18) | ZFJXT |

p<0.05 signifies statistical significance (marked in light yellow)

Table S2 BI League Table

| MD 95%CI | | | | | | | | | | | |
| --- | --- | --- | --- | --- | --- | --- | --- | --- | --- | --- | --- |
| BXBZTMT |  |  |  |  |  |  |  |  |  |  |  |
| 5.63 (-2.82, 14.14) | BYHWT |  |  |  |  |  |  |  |  |  |  |
| 4.7 (-4.24, 13.69) | -0.94 (-6.48, 4.57) | HTTLT |  |  |  |  |  |  |  |  |  |
| 16.92 (9.11, 24.72) | 11.27 (7.97, 14.56) | 12.22 (7.78, 16.63) | SOC |  |  |  |  |  |  |  |  |
| -3.82 (-16.19, 8.53) | -9.44 (-19.61, 0.64) | -8.5 (-19.05, 1.99) | -20.71 (-30.33, -11.13) | SQTLF |  |  |  |  |  |  |  |
| 7.15 (-3.24, 17.54) | 1.51 (-6.23, 9.22) | 2.47 (-5.84, 10.64) | -9.77 (-16.7, -2.82) | 10.97 (-0.85, 22.8) | TLFZT |  |  |  |  |  |  |
| 13.09 (2.67, 23.12) | 7.45 (-0.13, 14.67) | 8.4 (0.2, 16.21) | -3.83 (-10.65, 2.63) | 16.9 (4.99, 28.28) | 5.95 (-3.86, 15.33) | TQHXT |  |  |  |  |  |
| -1.74 (-10.99, 7.56) | -7.38 (-13.4, -1.36) | -6.43 (-13.11, 0.29) | -18.66 (-23.65, -13.59) | 2.08 (-8.76, 12.87) | -8.89 (-17.43, -0.32) | -14.83 (-22.94, -6.28) | XFZYT |  |  |  |  |
| 10.97 (1.15, 20.87) | 5.35 (-1.55, 12.2) | 6.28 (-1.16, 13.72) | -5.93 (-11.94, 0.07) | 14.79 (3.49, 26.1) | 3.84 (-5.32, 13.01) | -2.1 (-10.85, 7.1) | 12.73 (4.86, 20.56) | XLCQT |  |  |  |
| 10.08 (-2.65, 22.84) | 4.47 (-6.16, 14.98) | 5.41 (-5.62, 16.37) | -6.82 (-16.91, 3.22) | 13.91 (0.02, 27.74) | 2.96 (-9.34, 15.11) | -3 (-14.8, 9.3) | 11.84 (0.54, 23.02) | -0.9 (-12.61, 10.84) | XXMT |  |  |
| 6.69 (-2.64, 15.99) | 1.04 (-5.07, 7.11) | 1.98 (-4.82, 8.7) | -10.23 (-15.37, -5.14) | 10.49 (-0.37, 21.33) | -0.46 (-9.12, 8.13) | -6.4 (-14.61, 2.14) | 8.42 (1.21, 15.57) | -4.29 (-12.17, 3.54) | -3.4 (-14.67, 7.86) | YQHXT |  |
| -1.89 (-11.2, 7.41) | -7.54 (-13.5, -1.55) | -6.59 (-13.23, 0.09) | -18.81 (-23.78, -13.84) | 1.91 (-8.86, 12.73) | -9.06 (-17.56, -0.49) | -14.99 (-23.07, -6.44) | -0.16 (-7.23, 6.92) | -12.88 (-20.63, -5.03) | -11.99 (-23.16, -0.76) | -8.58 (-15.68, -1.41) | YQHXTLT |

Table S3 ADL League Table

| MD 95%CI | | | | | | | | | |
| --- | --- | --- | --- | --- | --- | --- | --- | --- | --- |
| BXBZTMT |  |  |  |  |  |  |  |  |  |
| -1.19 (-11.77, 9.05) | BYHWT |  |  |  |  |  |  |  |  |
| -18.38 (-36.83, -0.15) | -17.22 (-33.58, -0.59) | HTTLT |  |  |  |  |  |  |  |
| 9.09 (-0.25, 18.34) | 10.29 (5.67, 15.12) | 27.5 (11.72, 43.36) | SOC |  |  |  |  |  |  |
| -3.96 (-22.23, 14.21) | -2.77 (-19.14, 13.82) | 14.44 (-7.8, 36.72) | -13.08 (-28.8, 2.64) | TLFZT |  |  |  |  |  |
| -5.5 (-18.68, 7.55) | -4.32 (-14.64, 6.24) | 12.91 (-5.45, 31.24) | -14.62 (-23.92, -5.34) | -1.53 (-19.86, 16.74) | TLXFT |  |  |  |  |
| -7.42 (-22.3, 7.29) | -6.22 (-18.55, 6.19) | 11.02 (-8.64, 30.45) | -16.52 (-28.05, -5.08) | -3.45 (-22.9, 15.96) | -1.9 (-16.76, 12.75) | XFZYT |  |  |  |
| 3.18 (-15.53, 21.67) | 4.34 (-12.34, 21.2) | 21.56 (-0.88, 44.15) | -5.93 (-22.06, 10.15) | 7.13 (-15.41, 29.6) | 8.68 (-9.95, 27.22) | 10.54 (-9.12, 30.37) | XLCQT |  |  |
| 1.09 (-13.58, 15.62) | 2.28 (-9.83, 14.57) | 19.51 (0.06, 38.86) | -8.02 (-19.3, 3.18) | 5.03 (-14.34, 24.41) | 6.6 (-8.03, 21.23) | 8.5 (-7.54, 24.63) | -2.08 (-21.79, 17.55) | XXMT |  |
| -1.62 (-20.84, 17.32) | -0.43 (-17.68, 16.91) | 16.79 (-6.15, 39.6) | -10.73 (-27.46, 5.84) | 2.33 (-20.57, 25.28) | 3.87 (-15.24, 22.87) | 5.77 (-14.35, 25.96) | -4.8 (-27.99, 18.34) | -2.69 (-22.86, 17.34) | YQHXTLT |
