# Supplementary material for: Efficacy of different traditional Chinese medicine decoctions in the treatment of ischemic stroke: a network meta-analysis
Source: Front Pharmacol. 2024 Nov 1;15:1486458. doi: 10.3389/fphar.2024.1486458 (PMC11565597; doi:10.3389/fphar.2024.1486458)
Supplement: Supplementary file 3 [file DataSheet2.docx]

**Supplementary Material 2** Inclusion of 119 studies

**REFERENCE：**

(1-119)

1. Zhihui Z, Xiaokui Z, Xiuhong W. Evaluation of the Value of Adjuvant Treatment of Acute Ischemic Stroke with Hanxia Baijiu Tianma Tang. *Cardiovascular Disease Journal of Integrated Traditional Chinese and Western Medicine* (2017) 5(13):150-1. doi: 10.16282/j.cnki.cn11-9336/r.2017.13.108.

2. Qi Z. Evaluation of the Effect of Hanxia Baijiu Tianma Tang Combined with Olanzapine in the Treatment of Acute Ischemic Stroke. *The Journal of Medical Theory and Practice* (2019) 32(16):2550-1. doi: 10.19381/j.issn.1001-7585.2019.16.022.

3. Feng L. Observation on the Effect of Hanxia Baijutsu Tianma Tang Combined with Clopidogrel on Platelet Level and Neurological Function in Patients with Acute Ischemic Stroke. *Guizhou Medical Journal* (2023) 47(01):88-9.

4. Kaili F, Lei H. In Fluence of Banxia Baizhu Tianma Decoction Combined with Clopidogrel on Motor Function,Platelet Function,Serum Hs-Crp and Ps Levels of Patients with Acute Ischemic Stroke. *Journal of Emergency in Traditional Chinese Medicine* (2018) 27(03):422-5.

5. Yong T, Liang C, Chao Z, Wenbo C, Jin L, Xiaoling W. Clinical Effect of Banxia Baizhu Tianma Decoction Combined with Clopi-Dogrel in the Treatment of Acute Ischemic Stroke. *China Medical Herald* (2019) 16(35):78-81.

6. Feng L, Qiongyao W, Yue C, Yuhui W. Analysis of the Efficacy of Hanxia Baijutsu Tianma Tang Combined with Edaravone in Wind-Phlegm-Blocking Acute Ischemic Stroke. *Xinjiang Journal of Traditional Chinese Medicine* (2022) 40(01):8-10.

7. Qi T, Kuangyi L, Zhixin W, Shangming H, Xuewen W, Jingli C, et al. Banxia Baizhu Tianma Decoction with Edaravone in Treatment of 40 Cases of Acute Ischemic Stroke. *Banxia Baizhu Tianma Decoction with Edaravone in Treatment of 40 Cases of Acute Ischemic Stroke* (2017) 26(14):94-6. doi: 10.3969/j.issn.1007-8517.2017.14.zgmzmjyyzz201714035.

8. Ting L, Zhihua L. Clinical Efficacy of Hanxia Baijiu Tianma Tang Combined with Edaravone in the Treatment of Acute Ischemic Stroke. *Chinese Science and Technology Periodicals Database (Full Text Edition) Medicine and Hygiene* (2021) (11):2.

9. Jiefan Z, Cuixiang L. Clinical Efficacy of Hanxia Baijiu Tianma Tang Combined with Edaravone in the Treatment of Acute Ischemic Stroke. *Chinese Journal of Clinical Rational Drug Use* (2021) 14(21):3.

10. Xiaoqing X. Clinical Efficacy of Hanxia Baijiu Tianma Tang Combined with Edaravone in the Treatment of Acute Ischemic Stroke. *Nei Mongol Journal of Traditional Chinese Medicine* (2019) 38(08):118-9. doi: 10.16040/j.cnki.cn15-1101.2019.08.073.

11. Quan Z, Xiaoling Z, Chunlin T, Lejun L. Analysis of the Effect of the Combination of Hanxia Baijutsu Tianma Tang and Edaravone in the Treatment of Wind-Phlegm Blockage Type Acute Ischemic Stroke. *Health Care Today* (2021) 21(14):58-60.

12. Lin L, Lili Y, Zheng W, Changzai W, Wei Z. Effects of Tonifying Yang and Returning Five Soups on Inflammatory Factors, Oxidative Stress Indexes and Cerebral Nerve Function in Patients with Acute Ischemic Stroke. *Modern Journal of Integrated Traditional Chinese and Western Medicine* (2022) 31(10):1391-5.

13. Guangheng Z, Fang Z, Renbin L. Impact of Buyang Huanwu Decoction on C-Reactive Protein Level and Hemorheology of Acute Ischemic Stroke. *Liaoning Journal of Traditional Chinese Medicine* (2014) 41(07):1438-9. doi: 10.13192/j.issn.1000-1719.2014.07.053.

14. Guihua S, Rongsheng Y. Effects of Tonifying Yang and Returning Five Soups on Neurological Function and Homocysteine in Patients with Acute Ischemic Stroke. *Medical Information* (2015) 000(031):544-6.

15. Guangheng Z, Fang Z, Renbin L. Observation of Buyanghuanwu Decoction on Neurological Function and Homocysteine in Patients with Acute Is- Chemic Stroke. *Hebei Journal of Traditional Chinese Medicine* (2014) 36(04):544-6.

16. Mei W. Effect of Tonifying Yang and Restoring Wu Tang on Serum Homocysteine in Cerebral Infarction of Qi Deficiency and Blood Stasis Type. *Clinical Journal of Traditional Chinese Medicine* (2014) 26(08):793-5. doi: 10.16448/j.cjtcm.2014.08.011.

17. Hui X, Yanli Z, Hui Z, Jun L. Evaluation of the Therapeutic Effect of Tonifying Yang and Restoring Five Soups on Ischemic Stroke Patients During the Recovery Period. *World Journal of Integrated Traditional and Western Medicine* (2017) 12(08):1155-7+76. doi: 10.13935/j.cnki.sjzx.170832.

18. Yu W, Jie W. Effects of Tonifying Yang and Returning Five Soups on Neurological Function and Related Immune Factors in Patients with Ischemic Stroke. *World Journal of Integrated Traditional and Western Medicine* (2019) 14(07):1010-3. doi: 10.13935/j.cnki.sjzx.190731.

19. Xiaohong L, Jin'e H. Improvement Effect of Tonifying Yang Returning Five Soup on Ankle Dorsiflexion Disorder in Patients with Qi Deficiency and Blood Stasis Evidence During the Recovery Period of Ischemic Stroke. *Guizhou Medical Journal* (2024) 48(03):380-2.

20. Anxiang X, Conghui Y, Ying C, Feng X. Clinical Study of Buyang Huanwu Decoction on Early Neurological Deterioration in Acute Ischemic Stroke Patients. *Journal of Traditional Chinese Medicine University of Hunan* (2017) 37(12):1418-21.

21. Linfang L. Effect of Supplemental Treatment of Patients with Ischemic Stroke of Qi Deficiency and Blood Stasis Type by Replenishing Yang and Restoring Wu Tang. *Chinese Journal of Clinical Rational Drug Use* (2022) 15(09):64-7. doi: 10.15887/j.cnki.13-1389/r.2022.09.018.

22. Puyu G. Observation on the Efficacy of Auxiliary Treatment of Ischemic Stroke in Young and Middle-Aged People by Replenishing Yang Huiwu Tang. *Practical Clinical Journal of Integrated Traditional Chinese and Western Medicine* (2018) 18(08):60-2. doi: 10.13638/j.issn.1671-4040.2018.08.028.

23. Baojun S, Le T, Xianwei Q, Hui Z. Rehabilitation Effect of Different Proportions of Buyang Huanwu Decoction and Astragalus on Patients with Ischemic Stroke. *Clinical Journal of Traditional Chinese Medicine* (2023) 35(09):1788-92. doi: 10.16448/j.cjtcm.2023.0926.

24. Haiting M, Biren L, Zehui L. Clinical Observation on the Treatment of Ischemic Cerebral Stroke by Tonifying Yang and Restoring Five Soups Combined with Aspirin. *Journal of Hubei University of Chinese Medicine* (2016) 18(04):58-61.

25. Weiqiang C, Hong G, Xiaoping B, Xingning Z. Clinical Study of Using Buyang Huanwu Ddecoction Combined with Bolivar in the Treatment of Ischemic Stroke. *Journal of Sichuan Traditional Chinese Medicine* (2021) 39(01):133-7.

26. Tao L. Evaluation of Curative Effect of Buyang Huanwu Decoction Combined with Danhong Injection in the Treatment of Ischemic Stroke. *Gems of Health* (2018) 000(030):183.

27. Zhengyan S. Clinical Efficacy of Tonifying Yang and Returning Five Soups Combined with Danhong Injection in the Treatment of Ischemic Stroke. *Practical Clinical Journal of Integrated Traditional Chinese and Western Medicine* (2018) 18(06):58-9. doi: 10.13638/j.issn.1671-4040.2018.06.029.

28. Chi Z, Qi W. Effect of Buyang Huanwu Decoction Combined with Butylphthalide on Neurological Function and Prognosis of Patients with Ischemic Stroke. *Journal of Clinical Research* (2020) 37(12):3.

29. Chi Z, Qi W. The Effect of Tonifying Yang Huiwu Tang Combined with Butylphthalide Soft Capsule in the Treatment of Patients Recovering from Ischemic Stroke. *Medical Journal of Chinese People's Health* (2020) 32(15):96-7.

30. Tuming L, Ping Z, Ying W, Xiao W, Changde W, Shuwen T, et al. Clinical Observation on the Effect of Tonifying Yang Huiwu Tang Combined with Western Medicines on Platelet Aggregation Rate and Prognosis of Acute Large Artery Atherosclerotic Cerebral Infarction. *Jiangsu Journal of Traditional Chinese Medicine* (2018) 50(05):31-3.

31. Qingzhe L. Observation on the Effect of Tonifying Yang Huiwu Tang Combined with Western Medicines in Treating Ischemic Cerebral Stroke with Qi Deficiency and Blood Stasis Type. *Clinical Journal of Traditional Chinese Medicine* (2018) 30(10):1876-8. doi: 10.16448/j.cjtcm.2018.0575.

32. Zheng W, Lin L. Efficacy of Buyang Huanwu Decoction Combined with Western Medicine in the Treatment of Vascular Dementia after Ischemic Stroke and Its Effect on Serum Inflammatory Factors, Oxidative Stress Indexes and Vascular Endothelial Active Substances. *Modern Journal of Integrated Traditional Chinese and Western Medicine* (2022) 31(04):469-73+90.

33. Wei H, Bin Y, Yang C. Clinical Efficacy and Effects on Blood Lipids and Blood Rheology of Replenishing Yang and Restoring Five Soups Combined with Conventional Western Medicine in the Treatment of Acute Ischemic Stroke. *Clinical Journal of Chinese Medicine* (2019) 11(10):56-8.

34. Fei M, Tao C, Yidong D, Yanhui L, Xia P, Yingying Z. Effects of Tonifying Yang and Restoring Five Soups Combined with Edaravone on Blood Rheology and Neurological Function in Patients with Ischemic Stroke. *Chinese Journal of Gerontology* (2018) 38(12):2824-6.

35. Fangfang Z, Chenglong W, Xiaohong G, Yiping L, Zhaowei W. Clinical Efficacy and Safety Evaluation of Buyang Huanwu Decoction Com-Bined with Edaravone in the Treatment of Acute Ischemic Stroke. *China Modern Doctor* (2021) 59(25):51-4.

36. Huishan L, Liu J, Yingli Z. Effect of Buyang Huanwu Decoction Combined with Edaravone in Treatment of Ischemic Stroke and Its Effect on Blood Rheology. *Chinese Archives of Traditional Chinese Medicine* (2016) 34(03):721-3. doi: 10.13193/j.issn.1673-7717.2016.03.062.

37. Zhengyin X. Influence of Tonifying Yang and Returning Five Soups Together with Western Medicine Treatment on the Rehabilitation Effect of Ischemic Stroke. *Clinical Journal of Chinese Medicine* (2020) 12(11):12-4.

38. Changhao Y, Chunxia D, Forman Z. The Effect of Five Decoction Combined with Western Medicine in Treatment of Ischemic Stroke:A Controlled Study. *Journal of Practical Traditional Chinese Internal* (2012) 26(17):49-50.

39. Yinghong L. Clinical Effects of Buyang Huanwu Decoction Combined with Edaravone Injection in Treating 48 Patients with Acute Ischemic Stroke. *Chinese Journal of Experimental Traditional Medical Formulae* (2013) 19(13):304-7.

40. Guibing Z, Xiaoke Z. Treatment of 40 Cases of Acute Ischemic Stroke with Tonifying Yang and Restoring Five Soups. *Henan Traditional Chinese Medicine* (2014) 34(10):2.

41. Tianhan Z, Tianchu C. Clinical Evaluation of Buyang Huanwu Decoction in the Treatment of Acute Ischemic Stroke and Its Influence on Nerve Function and Hemorheology. *World Latest Medicine Information* (2023) 23(45):99-102,11. doi: 10.3969/j.issn.1671-3141.2023.045.019.

42. Hongsheng H. Clinical Efficacy of Tonifying Yang and Restoring Wu Tang in Treating Patients with Acute Ischemic Stroke and Its Effect on Inflammatory Response and Blood Rheology. *Chinese Journal of Clinical Rational Drug Use* (2022) 15(09):71-3. doi: 10.15887/j.cnki.13-1389/r.2022.09.020.

43. Ailing C, Congcong M, Cunyong L. Clinical Efficacy of Buyang Huanwu Decoction in the Treatment of Stroke and Influence on Hemorheology. *World Chinese Medicine* (2018) 13(03):628-31.

44. Zhiqiang H, Zhaoxia C, Xiaojuan C, Qisheng H, Zhi D, Longhai W, et al. Clinical Application and Mechanism of Buyang Huanwu Decoction in Acute Ischemic Stroke Patients with Qi Deficiency Blood Stasis Syndrome. *Chinese Journal of Integrated Traditional and Western Medicine* (2022) 42(1):24-8. doi: 10.7661/j.cjim.20210106.152.

45. Liangtianjiao L. Efficacy Observation on 46 Cases of Ischemic Stroke Treated by Replenishing Yang and Returning Five Soups. *Guide of China Medicine* (2012) 10(16):270-1.

46. Wucheng W. Therapeutic Efficacy and Effect on Neurological Function of Replenishing Yang Huiwu Tang in the Treatment of Ischemic Stroke. *Psychologies Magazine* (2020) (11):1.

47. Meiling C, Chunjuan Z. Effects of Buyang Huanwu Decoction on Patients with Ischemic Stroke. *Medical Journal of Chinese People's Health* (2022) 34(12):109-11+15.

48. Shi J, Jing Z, Kegia Po. Effect of Shenqi Tongluo Decoction Combined Rehabilitation Training on Neurological Function and Disability Rate in Hemiplegia Patients with Ischemic Stroke. *Journal of Liaoning University of Traditional Chinese Medicine* (2019) 21(07):209-12. doi: 10.13194/j.issn.1673-842x.2019.07.055.

49. Rongrong L. Effect of Shenqi Tongluo Decoction Combined with Western Medicine on Patients with Hemiplegia after Stroke. *Medical Journal of Chinese People's Health* (2022) 34(19):84-7.

50. Hua H. Efficacy of Ginseng Qi Tongluo Soup in the Treatment of Ischemic Stroke Hemiplegia During the Recovery Period and Its Effect on Limb Function.

51. Xiaoyan Z, Yan LL, Maoqing W. Influence of Huatan Tongluo Decoction, Acupuncture Plus Rehabilitation Training on Neurological Function for Stroke Patients. *Contemporary Medicine* (2020) 26(22):3.

52. Tan F, Bornan H. Effects of Resolving Phlegm and Penetrating Tang on Neurological Function and Activities of Daily Living of Patients in the Acute Stage of Ischemic Stroke. *Guiding Journal of Traditional Chinese Medicine and Pharmacology* (2013) 19(5):2.

53. Zhenyong P. Observations on the Therapeutic Effect of Wind-Phlegm-Blocking Type of Acute Ischemic Cerebral Stroke Aided by Resolving Phlegm and Clearing Collaterals Soup. *Journal of Practical Traditional Chinese Medicine* (2020) 36(7):2.

54. Xiangqian X, Yanbing D. Observation on the Clinical Effect of Huatan Tongluo Decoction Combined with Intravenous Thrombolysis on Acute Ischemic Stroke. *World Latest Medicine Information* (2019).

55. Shiyong L, Yuanyang C, Qizhi W, Ching FK, Kei LO. Effects of Huatan Tongluo Decoction Combined with Routine Western Medicine on Neurological Function, Platelet Function and Serum Inflammatory Factors in Patients with Ischemic Stroke. *Progress in Modern Biomedicine* (2020) 20(12):4.

56. Qiuying Z, Yanyan L. Clinical Study on Huatan Tongluo Tang Combined with Rehabilitation Training for Acute Ischemic Stroke. *Journal of New Chinese Medicine* (2021).

57. Qingsan S. Clinical Effects of Huatan Tongluo Decoction Combined with Edaravone in the Treatment of Ischemic Stroke with Wind Phlegm Obstructing Collaterals Type. *Clinical Research and Practice* (2018) 3(16):137-8. doi: 10.19347/j.cnki.2096-1413.201816065.

58. Shinian Z. The Effect of Huatan Tongluo Decoction Combined with Acupuncture on Sequela of Stroke. *China Continuing Medical Education* (2019) 11(8):3.

59. Zhijiang Z, Lihong Z, Chongchong L. Observation on the Therapeutic Effect of Resolving Phlegm and Clearing Collaterals in the Treatment of Ischemic Stroke Patients and Its Effect on Serum Nse,Hcy. *Hubei Journal of Traditional Chinese Medicine* (2022).

60. Qing L, Biaoxin C, Lin L, Zhiqiang L. Application Effect of Tonifying Yang Huiwu Tang in the Treatment of Acute Ischemic Stroke with Qi Deficiency and Blood Stasis Evidence. *Journal of Chinese and Foreign Medical Research* (2023) 2(12):190-3. doi: 10.12417/2811-051x.23.12.064.

61. Zhiqing S. Dispelling Pathogenic Wind and Expelling Blood Stasis for Promoting Blood Circulation and Dredging Collateral Prescription in Treating Cerebral Ischemic Stroke for 42 Cases. *Guangming Journal of Chinese Medicine* (2017) 32(10):1456-7.

62. Hongmin S, Hongying M, Xiaolin W. Effects of the Formula of Dispelling Wind, Resolving Blood Stasis, Promoting Blood Circulation and Clearing Collaterals on Neurological Function and Platelets in Patients with Ischemic Stroke. *Chinese Journal of Integrative Medicine on Cardio/Cerebrovascular Disease* (2015) 13(11):1317-9.

63. Renjie Z. Effect of "Qufeng Xingxue Tongluo Formula" on Platelet Function of Patients with Ischemic Stroke. *Shanghai Journal of Traditional Chinese Medicine* (2015) 49(03):49-51. doi: 10.16305/j.1007-1334.2015.03.007.

64. Jinpeng Y. Effect of Buyang Huanwu Decoction Combined with Western Medicine on Nerve Function Recovery and Carotid Atherosclerotic Plaque in Patients with Ischemic Stroke. *World Journal of Complex Medicine* (2023) 9(06):146-8+52.

65. Guopeng C, Heyu J. Clinical Efficacy of Tongluo Fuzheng Tang on Early Intervention of Hemiplegia in Elderly Ischemic Stroke. *Guizhou Medical Journal* (2023).

66. Jia S. Clinical Efficacy of Tongluo Fuzheng Tang Combined with Acupuncture in the Treatment of Patients with Hemiplegia after Ischemic Stroke. *New Mom and New Born* (2021) (8):98.

67. Yan C, Juguang S, Guanghui J, Qian S. Effect Analysis of Tongluo Fuzheng Decoction Combined with Acupunc?Ture in Treating Hemiplegic Patients after Ischemic Stroke. *Systems Medicine* (2022) 7(19):44-7. doi: 10.19368/j.cnki.2096-1782.2022.19.044.

68. Yanli S. Effect of Tongluo Xifeng Decoction on Nerve Function and Hemody-Namics in Patients with Ischemic Stroke. *Systems Medicine* (2022) 7(08):195-8. doi: 10.19368/j.cnki.2096-1782.2022.08.195.

69. Xujie C. 90 Cases of Acute Ischemic Stroke Treated with Tongluo and Wind Quenching Soup Combined with Conventional Therapy. *Traditional Chinese Medicinal Research* (2018) 31(6):3.

70. Renfeng H, Qing S, Xiaoliang L, Jieming C, Ronghao C, Tao Y. Clinical Efficacy Observation of Tongluo Quenching Tang Combined with Western Basic Therapy on Acute Stage of Ischemic Stroke and Its Safety Evaluation. *Guangzhou Medical Journal* (2016) 47(1):3.

71. Jian L. Study on the Clinical Efficacy of Tongluo Quenching Tang in Treating Patients with Ischemic Stroke in the Acute Phase. *Chinese Science and Technology Periodicals Database (Full Text Edition) Medicine and Hygiene* (2021) (10):2.

72. Zhang L, Bo S. The Effect of Tongkou Xiebao Tang Combined with Aspirin on Oxidative Stress and the Degree of Neurological Deficits in Patients with Ischemic Stroke. *Guiding Journal of Traditional Chinese Medicine and Pharmacology* (2017) 23(16):3.

73. Ying J, Hui L, Xing F, Yongquan Z. Clinical Observation on Tongqiao Huoxue Tang Combined with Aspirin for Ischemic Stroke. *Chinese Journal of Experimental Traditional Medical Formulae* (2016) 22(8):4.

74. Yajing L. Efficacy and Effect on Vegf in Acute Ischemic Stroke Treated with Tongkou Xiexue Tang Combined with Butylphthalide. *Special Health* (2022) (6):64-6.

75. Sufang M, Yu W, Chen J. Clinical Study on Tongqiao Huoxue Tang Combined with Brain-Awakening and Orifices-Opening Acupuncture for Ischemic Stroke. *Journal of New Chinese Medicine* (2022) (014):054.

76. Xue L, Jingjun J. Effects of Tongqiao Huoxue Decoction Combined with Tcm Rehabilitation on Neurological Function Recovery in Stroke Patients. *Journal of Hubei University of Chinese Medicine* (2022).

77. Zuchao J, Wenzong Z, Feiwen L, Yongxi J. Effect of Xiaoxuming Decoction on Neurological Function and Tcm Syn-Drome of Patients with Acute Ischemic Stroke. *China Modern Doctor* (2019) 57(5):4.

78. Xianjun L, Chuiyi Z. Study on the Efficacy of Xiao Renmin Tang on Ischemic Stroke and Its Effect on Neurological Function and Blood Rheology. *Asia-Pacific Traditional Medicine* (2018) 14(4):3.

79. Shaojun A, Min W, Fang G. Analysis of the Efficacy of the Treatment of Ischemic Stroke with the Addition and Subtraction of Xiao Renmin Tang Combined with Acupuncture. *Modern Medicine and Health Research* (2022) 6(11):4.

80. Qing Z. Analysis of the Clinical Effect of Small Life-Sustaining Soup Plus Reduction Combined with Acupuncture in the Treatment of Ischemic Stroke Patients. *Modern Diagnosis & Treatment* (2023) 34(16):2389-91.

81. Li L, Ocean S, Yanling G. Analysis of Therapeutic Efficacy of the Addition and Subtraction of Xiao Renmin Tang in the Treatment of Acute Ischemic Stroke (Wind-Fire Upheaval Syndrome). *Journal of Emergency in Traditional Chinese Medicine* (2020) 29(4):4.

82. Lei Z, Yi H, Feng X. Effect of Xinglou Chengqi Decoction on Perioperative Prognosis of Intravascu-Lar Treatment for Acute Ischemic Stroke. *Journal of Hubei University of Chinese Medicine* (2023) 25(6):62-5.

83. Ji M. Therapeutic Efficacy of Xingguo Chengqi Tang Combined with Aspirin in the Treatment of Acute Ischemic Stroke. *Shenzhen Journal of Integrated Traditional Chinese and Western Medicine* (2020) (15):3.

84. Qiang Z. Clinical Effect Analysis of Xingguo Chengqi Tang in Treating Patients with Acute Stage of Ischemic Stroke. *Journal of Community Medicine* (2017) 15(8):2.

85. Hongwei L, Liping W. Clinical Effect Analysis of Xinglou Chengqi Decoction in Treating Acute Stage of Ischemic Stroke. *Clinical Journal of Traditional Chinese Medicine* (2020) 32(05):931-3. doi: 10.16448/j.cjtcm.2020.0531.

86. Shuhui Y, Shuang L, Huanmin N, Shuyun L. Clinical Observation on Xinglou Chengqi Decoction in the Treatment of Acute Ischemic Stroke with Phlegm-Heat Fu-Sthenia Syndrome. *Chinese Journal of Integrative Medicine on Cardio/Cerebrovascular Disease* (2018) 16(11):1487-9.

87. Jia D. Effect of Blood-Fu and Blood-Stasis-Expelling Soup on Motor Function in Patients with Spastic Paralysis after Ischemic Stroke. *Journal of North Pharmacy* (2020) 17(11):120-1.

88. Haili L, Ran W, Xiang W, Cao G. Effects of Xuefu Zhuyu Decoction on Motor Function in Patients with Spastic Paralysis after Ischemic Stroke. *Hebei Journal of Traditional Chinese Medicine* (2018) 40(6):5.

89. Huifen G, Yanmei H, Wei W, Shumin Y, Sumei C, Xiaofeng C, et al. Effect of Xuefu Zhuyu Decoction on Ischemic Stroke Hemiplegia. *Liaoning Journal of Traditional Chinese Medicine* (2022) 49(10):4.

90. Hua Y. The Effects of the Flavored Formula of Blood Palace and Blood Stasis Tang on Neurological Scores and Sleep Quality of Patients with Post-Stroke Depression. *Modern Journal of Integrated Traditional Chinese and Western Medicine* (2020) 29(28):4.

91. Dajun T, Yajun T. Observation on the Effect of Treating Acute Ischemic Stroke by Combining Bloodfu and Blood Stasis Soup with Western Medicines. *Clinical Journal of Chinese Medicine* (2016) 8(27):2.

92. Keyun F. Therapeutic Effect of Blood-Fu and Blood-Stasis-Expelling Soup Combined with Butylphthalide in the Treatment of Acute Ischemic Stroke. *Chinese Journal of Geriatric Care* (2020) 18(3):3.

93. Minghui D. The Effect of Blood-Fu and Blood-Stasis-Expelling Soup Combined with Western Medicine Treatment on the Neurological Function of Patients with Acute Ischemic Stroke. *Medical Journal of Chinese People's Health* (2018) 30(22):3.

94. Jiang X, Kunlin Z. Effectiveness of Blood-Fu and Blood-Stasis-Expelling Soup Combined with Acupuncture in the Treatment of Patients with Ischemic Stroke. *Gems of Health* (2023) (7):183-4.

95. Fei W, Jing T. Xuefu Zhuyu Decoction for Acute Ischemic Stroke. *China Journal of Chinese Medicine* (2019).

96. Yinghong L. Therapeutic Efficacy of Hematopoietic and Blood-Stasis-Expelling Soup in the Treatment of Acute Ischemic Stroke. *Chinese Journal of Rehabilitation Theory and Practice* (2013) (3):263-5.

97. GAOHong, DANGLi-li, LIJian-jun. Clinical Effect of Xuefu Zhuyu Decoction in Acute Ischemic Stroke and Its Effect on High Sensitivity C Reactive Protein and Hemorheology. *Hainan Medical Journal* (2017) 28(22):3.

98. Jian L, Yunquan D. Efficacy of Blood-Fu and Blood-Stasis-Expelling Soup in Treating Acute Ischemic Stroke and Its Effect on Serum Inflammatory Factor and Blood Rheology. *Shaanxi Journal of Traditional Chinese Medicine* (2017) 38(10):2.

99. Dongshan L. Treatment of Ischemic Stroke in the Elderly with Hemofluorescence and Its Effect on Patients' Hemorheology and Neurological Function by Hemofluorescence and Blood Stasis Soup. *Gansu Science and Technology* (2020) 36(23):4.

100. Weidong Z. Treatment of Ischemic Stroke by Blood Operator by Stasis Decoction. *Jilin Journal of Traditional Chinese Medicine* (2015) (9):4.

101. Chengwei Z. Clinical Study on the Treatment of Acute Ischemic Stroke by Combining Western Medicines with Blood Vessel and Blood Stasis Tang. *Asia-Pacific Traditional Medicine* (2015) 11(21):2.

102. Xianghong T. The Effect of Thrombotoxin Combined with Tonic Yang Returning Five Soup on Nihss and Blood Rheology Indexes of Ischemic Stroke Patients. *Chinese Journal of Gerontology* (2014).

103. Sun S. Effects of Yiqi and Blood Activation Soup on Neurological Function and Blood Rheology of Ischemic Stroke Patients. *Shaanxi Journal of Traditional Chinese Medicine* (2015) (7):2.

104. Zihua G. Effects of Yiqi and Blood-Boosting Soup Combined with Butylphthalide on Serum Ua,Et-1,Ptx-3,Sicam-1 and Neurological Function in Patients with Ischemic Stroke. *Chinese Journal of Integrative Medicine on Cardio-Cerebrovascular Disease* (2018) 16(8):4.

105. Haitao L, Yuping X. Clinical Observation on the Treatment of Ischemic Stroke Sequelae with Qi Deficiency and Blood Stasis Type by Combining Yiqi and Blood Activation Soup with Rehabilitation Training. *Guangming Journal of Chinese Medicine* (2021) 36(12):4.

106. Junfeng X. Effect of Yiqi Huoxue Decoction Combined with Western Medicine on Nerve Function and Carotid Atherosclerotic Plaque in Patients with Ischemic Stroke. *Medical Innovation of China* (2020) 17(36):4.

107. Fan Y, Xiaoping W. Effects of Yiqi and Blood-Activating Soup Combined with Systematic Nursing Intervention Model on Neurological Function and Serum Level of Ischemic Stroke Patients. *Journal of Yanan University(Medical Sciences)* (2020) 18(4):3.

108. Chunxia Z, Luming Z, Xueying Q, Hongfeng W, Peifu W. Efficacy of Yiqi Huoxue Decoction on Ischemic Stroke and Its Effect on Platelet Aggregation. *Journal of Emergency in Traditional Chinese Medicine* (2022) (006):031.

109. Hong L. Clinical Observation on the Treatment of Qi Deficiency and Blood Stasis During the Recovery Period of Stroke with the Formula of Benefiting Qi, Activating Blood and Clearing Collaterals. *Shenzhen Journal of Integrated Traditional Chinese and Western Medicine* (2018) 28(19):3.

110. Ping FY, Xiaodong J. Clinical Observation of Yiqi Huoxue Tongluo Tang Combined with Western Medicine for Ischemic Stroke in Recovery Stage of Qi Deficiency and Blood Stasis Type. *Journal of New Chinese Medicine* (2018) 50(3):4.

111. Xunming G, Xiaosong T. Clinical Analysis of Yiqi Huoxue Tongluo Decoction in the Treatment of Ischemic Cerebral Apoplexy. *World Chinese Medicine* (2018) 13(11):4.

112. Changming D, Jiacheng Z. Clinical Efficacy of Yiqi Huoxue Tongluo Decoction in the Treatment of Patients with Ischemic Stroke. *Clinical Research and Practice* (2018) 3(7):2.

113. Xiaoqing L, Qian Z, Yi S. Effectiveness of Yiqi, Blood Activation and Collateralization Tang in Treating Patients with Ischemic Stroke in the Recovery Period and Its Effect on Hemodynamic Levels and No, Vegf and Et-1 Levels. *Journal of Sichuan Traditional Chinese Medicine* (2019) 37(2):3.

114. Shaopeng Z. Observation on the Effect of Benefiting Qi, Activating Blood and Promoting Collaterals in the Treatment of Ischemic Stroke by Tongluo Tang. *Chinese Journal of Clinical Rational Drug Use* (2019) 12(19):67-9. doi: 10.15887/j.cnki.13-1389/r.2019.19.034.

115. Chunfeng G, Zhirong Y. Therapeutic Efficacy of Qi-Benefiting and Blood-Activating Traditional Chinese Medicine in the Treatment of Hemorrhagic Stroke During the Acute Stage of Hemorrhagic Stroke. *Practical Clinical Journal of Integrated Traditional Chinese and Western Medicine* (2008) 8(3):2.

116. Hairong H, Dequan L. The Effect of Zhongfeng Jiuxian Decoction with Butylphthalide Soft Capsule in the Treatment of Progressive Isch-Emic Stroke on the Degree of Neurologic Function Impairment. *HEILONG MEDICAL JOURANL* (2021).

117. Xiaochun S. Clinical Effect of Stroke Rescue Soup in the Treatment of Ischemic Progressive Stroke. *Heilongjiang Journal of Traditional Chinese Medicine* (2018) 47(5):2.

118. Xiaoming P, Xiaoqing Y, Weiming W, Shihuo C. Clinical Observation on Zhongfeng Jiuxian Decoction in the Treatment of Progressive Ischemic Stroke. *Chinese Journal of Integrative Medicine on Cardio-Cerebrovascular Disease* (2018) 16(7):4.

119. Liang S, Wu Y, Zhang R, Xin X, Xu L. Therapeutic Effects of Buyang Huanwu Tang Combined with Rt-Pa Intravenous Thrombolysis on Stroke of Qi Deficiency and Blood Stasis Type and Its Impact on Keap1-Nrf2/Are Pathway Antioxidant Stress. *Cell Mol Biol (Noisy-le-grand)* (2023) 69(13):210-6. Epub 2024/01/02. doi: 10.14715/cmb/2023.69.13.32.
