# Supplementary material for: Efficacy of different traditional Chinese medicine decoctions in the treatment of ischemic stroke: a network meta-analysis
Source: Front Pharmacol. 2024 Nov 1;15:1486458. doi: 10.3389/fphar.2024.1486458 (PMC11565597; doi:10.3389/fphar.2024.1486458)
Supplement: Supplementary file 4 [file DataSheet1.docx]

**Supplementary Material 1** Search strategy

PUBMED

((((Decoction[Title/Abstract]) OR (Soup[Title/Abstract])) OR (Tang[Title/Abstract])) AND ((Ischemic Stroke[MeSH Terms]) OR (((((((((((((((((((((((((Ischemic Stroke[Title/Abstract]) OR (Ischemic Strokes[Title/Abstract])) OR (Stroke, Ischemic[Title/Abstract])) OR (Ischaemic Stroke[Title/Abstract])) OR (Ischaemic Strokes[Title/Abstract])) OR (Stroke, Ischaemic[Title/Abstract])) OR (Cryptogenic Ischemic Stroke[Title/Abstract])) OR (Cryptogenic Ischemic Strokes[Title/Abstract])) OR (Ischemic Stroke, Cryptogenic[Title/Abstract])) OR (Stroke, Cryptogenic Ischemic[Title/Abstract])) OR (Cryptogenic Stroke[Title/Abstract])) OR (Cryptogenic Strokes[Title/Abstract])) OR (Stroke, Cryptogenic[Title/Abstract])) OR (Cryptogenic Embolism Stroke[Title/Abstract])) OR (Cryptogenic Embolism Strokes[Title/Abstract])) OR (Embolism Stroke, Cryptogenic[Title/Abstract])) OR (Stroke, Cryptogenic Embolism[Title/Abstract])) OR (Wake-up Stroke[Title/Abstract])) OR (Stroke, Wake-up[Title/Abstract])) OR (Wake up Stroke[Title/Abstract])) OR (Wake-up Strokes[Title/Abstract])) OR (Acute Ischemic Stroke[Title/Abstract])) OR (Acute Ischemic Strokes[Title/Abstract])) OR (Ischemic Stroke, Acute[Title/Abstract])) OR (Stroke, Acute Ischemic[Title/Abstract])))) AND (randomized controlled trial[Publication Type] OR randomized[Title/Abstract] OR placebo[Title/Abstract])

EMBASE

| No. | Query | Results | Date |
| --- | --- | --- | --- |
| #37 | #32 AND #36 | 11 | 17-Apr-24 |
| #36 | #33 OR #34 OR #35 | 890579 | 17-Apr-24 |
| #35 | 'double-blind':ab,ti | 230139 | 17-Apr-24 |
| #34 | 'placebo':ab,ti | 375717 | 17-Apr-24 |
| #33 | 'random':ab,ti | 454639 | 17-Apr-24 |
| #32 | #27 AND #31 | 214 | 17-Apr-24 |
| #31 | #28 OR #29 OR #30 | 24184 | 17-Apr-24 |
| #30 | 'tang':ab,ti | 6459 | 17-Apr-24 |
| #29 | 'soup':ab,ti | 3671 | 17-Apr-24 |
| #28 | 'decoction':ab,ti | 14609 | 17-Apr-24 |
| #27 | #1 OR #2 OR #3 OR #4 OR #5 OR #6 OR #7 OR #8 OR #9 OR #10 OR #11 OR #12 OR #13 OR #14 OR #15 OR #16 OR #17 OR #18 OR #19 OR #20 OR #21 OR #22 OR #23 OR #24 OR #25 OR #26 | 141130 | 17-Apr-24 |
| #26 | 'stroke, acute ischemic':ab,ti | 53 | 17-Apr-24 |
| #25 | 'ischemic stroke, acute':ab,ti | 201 | 17-Apr-24 |
| #24 | 'acute ischemic strokes':ab,ti | 877 | 17-Apr-24 |
| #23 | 'acute ischemic stroke':ab,ti | 39225 | 17-Apr-24 |
| #22 | 'wake-up strokes':ab,ti | 187 | 17-Apr-24 |
| #21 | 'wake up stroke':ab,ti | 501 | 17-Apr-24 |
| #20 | 'stroke, wake-up':ab,ti | 23 | 17-Apr-24 |
| #19 | 'wake-up stroke':ab,ti | 501 | 17-Apr-24 |
| #18 | 'stroke, cryptogenic embolism':ab,ti | 0 | 17-Apr-24 |
| #17 | 'embolism stroke, cryptogenic':ab,ti | 0 | 17-Apr-24 |
| #16 | 'cryptogenic embolism strokes':ab,ti | 0 | 17-Apr-24 |
| #15 | 'cryptogenic embolism stroke':ab,ti | 1 | 17-Apr-24 |
| #14 | 'stroke, cryptogenic':ab,ti | 26 | 17-Apr-24 |
| #13 | 'cryptogenic strokes':ab,ti | 551 | 17-Apr-24 |
| #12 | 'cryptogenic stroke':ab,ti | 3721 | 17-Apr-24 |
| #11 | 'stroke, cryptogenic ischemic':ab,ti | 0 | 17-Apr-24 |
| #10 | 'ischemic stroke, cryptogenic':ab,ti | 9 | 17-Apr-24 |
| #9 | 'cryptogenic ischemic strokes':ab,ti | 43 | 17-Apr-24 |
| #8 | 'cryptogenic ischemic stroke':ab,ti | 406 | 17-Apr-24 |
| #7 | 'stroke, ischaemic':ab,ti | 281 | 17-Apr-24 |
| #6 | 'ischaemic strokes':ab,ti | 1342 | 17-Apr-24 |
| #5 | 'ischaemic stroke':ab,ti | 15097 | 17-Apr-24 |
| #4 | 'stroke, ischemic':ab,ti | 1264 | 17-Apr-24 |
| #3 | 'ischemic strokes':ab,ti | 7993 | 17-Apr-24 |
| #2 | 'ischemic stroke':ab,ti | 111807 | 17-Apr-24 |
| #1 | 'ischemic stroke'/exp | 33673 | 17-Apr-24 |

WEB OF SCIENCE：

| Entitlements | # | Search Query | Database | Results | Date Run |
| --- | --- | --- | --- | --- | --- |
| - WOS.IC: 1993 to 2024 - WOS.CCR: 1985 to 2024 - WOS.SCI: 1975 to 2024 - WOS.AHCI: 1975 to 2024 - WOS.BHCI: 2005 to 2024 - WOS.BSCI: 2005 to 2024 - WOS.ESCI: 2019 to 2024 - WOS.ISTP: 1990 to 2024 - WOS.SSCI: 1965 to 2024 - WOS.ISSHP: 1990 to 2024 | 1 | TS=(Ischemic Stroke) OR TS=(Ischemic Strokes) OR TS=(Stroke, Ischemic) OR TS=(Ischaemic Stroke) OR TS=(Ischaemic Strokes) OR TS=(Stroke, Ischaemic) OR TS=(Cryptogenic Ischemic Stroke) OR TS=(Cryptogenic Ischemic Strokes) OR TS=(Ischemic Stroke, Cryptogenic) OR TS=(Stroke, Cryptogenic Ischemic) OR TS=(Cryptogenic Stroke) OR TS=(Cryptogenic Strokes) OR TS=(Stroke, Cryptogenic) OR TS=(Cryptogenic Embolism Stroke) OR TS=(Cryptogenic Embolism Strokes) OR TS=(Embolism Stroke, Cryptogenic) OR TS=(Stroke, Cryptogenic Embolism) OR TS=(Wake-up Stroke) OR TS=(Stroke, Wake-up) OR TS=(Wake up Stroke) OR TS=(Wake-up Strokes) OR TS=(Acute Ischemic Stroke) OR TS=(Acute Ischemic Strokes) OR TS=(Ischemic Stroke, Acute) OR TS=(Stroke, Acute Ischemic) | Web of Science Core Collection | 150789 | Wed Apr 17 2024 22:38:28 GMT+0800 |
| - WOS.IC: 1993 to 2024 - WOS.CCR: 1985 to 2024 - WOS.SCI: 1975 to 2024 - WOS.AHCI: 1975 to 2024 - WOS.BHCI: 2005 to 2024 - WOS.BSCI: 2005 to 2024 - WOS.ESCI: 2019 to 2024 - WOS.ISTP: 1990 to 2024 - WOS.SSCI: 1965 to 2024 - WOS.ISSHP: 1990 to 2024 | 2 | TS=(Decoction) OR TS=(Soup) OR TS=(Tang) | Web of Science Core Collection | 27923 | Wed Apr 17 2024 22:38:37 GMT+0800 |
| - WOS.IC: 1993 to 2024 - WOS.CCR: 1985 to 2024 - WOS.SCI: 1975 to 2024 - WOS.AHCI: 1975 to 2024 - WOS.BHCI: 2005 to 2024 - WOS.BSCI: 2005 to 2024 - WOS.ESCI: 2019 to 2024 - WOS.ISTP: 1990 to 2024 - WOS.SSCI: 1965 to 2024 - WOS.ISSHP: 1990 to 2024 | 3 | TS=(random) OR TS=(placebo) OR TS=(double-blind) OR TS=(randomized controlled trial) OR TS=(randomized) | Web of Science Core Collection | 2255451 | Wed Apr 17 2024 22:38:44 GMT+0800 |
| - WOS.IC: 1993 to 2024 - WOS.CCR: 1985 to 2024 - WOS.SCI: 1975 to 2024 - WOS.AHCI: 1975 to 2024 - WOS.BHCI: 2005 to 2024 - WOS.BSCI: 2005 to 2024 - WOS.ESCI: 2019 to 2024 - WOS.ISTP: 1990 to 2024 - WOS.SSCI: 1965 to 2024 - WOS.ISSHP: 1990 to 2024 | 4 | #3 AND #2 AND #1 | Web of Science Core Collection | 34 | Wed Apr 17 2024 22:38:59 GMT+0800 |

COCHRANCE：

ID Search Hits

#1 MeSH descriptor: [Ischemic Stroke] explode all trees 1456

#2 (Ischemic Stroke):ti,ab,kw OR (Ischemic Strokes):ti,ab,kw OR (Stroke, Ischemic):ti,ab,kw OR (Ischaemic Stroke):ti,ab,kw OR (Ischaemic Strokes):ti,ab,kw 20059

#3 (Stroke, Ischaemic):ti,ab,kw OR (Cryptogenic Ischemic Stroke):ti,ab,kw OR (Cryptogenic Ischemic Strokes):ti,ab,kw OR (Ischemic Stroke, Cryptogenic):ti,ab,kw OR (Stroke, Cryptogenic Ischemic):ti,ab,kw 19915

#4 (Cryptogenic Stroke):ti,ab,kw OR (Cryptogenic Strokes):ti,ab,kw OR (Stroke, Cryptogenic):ti,ab,kw OR (Cryptogenic Embolism Stroke):ti,ab,kw OR (Cryptogenic Embolism Strokes):ti,ab,kw 279

#5 (Embolism Stroke, Cryptogenic):ti,ab,kw OR (Stroke, Cryptogenic Embolism):ti,ab,kw OR (Wake-up Stroke):ti,ab,kw OR (Stroke, Wake-up):ti,ab,kw OR (Wake up Stroke):ti,ab,kw 327

#6 (Wake-up Strokes):ti,ab,kw OR (Acute Ischemic Stroke):ti,ab,kw OR (Acute Ischemic Strokes):ti,ab,kw OR (Ischemic Stroke, Acute):ti,ab,kw OR (Stroke, Acute Ischemic):ti,ab,kw 9959

#7 #1 or #2 or #3 or #4 or #5 or #6 20328

#8 (Decoction):ti,ab,kw OR (Soup):ti,ab,kw OR (Tang):ti,ab,kw 7333

#9 #7 and #8 53
